# A Bayesian model based computational analysis of the relationship between bisulfite accessible single-stranded DNA in chromatin and somatic hypermutation of immunoglobulin genes

**DOI:** 10.1371/journal.pcbi.1009323

**Published:** 2021-09-07

**Authors:** Guojun Yu, Yingru Wu, Zhi Duan, Catherine Tang, Haipeng Xing, Matthew D. Scharff, Thomas MacCarthy

**Affiliations:** 1 Department of Cell Biology, Albert Einstein College of Medicine, Bronx, New York, United States of America; 2 Department of Applied Mathematics and Statistics, Stony Brook University, Stony Brook, New York, United States of America; Ecole normale superieure, FRANCE

## Abstract

The B cells in our body generate protective antibodies by introducing somatic hypermutations (SHM) into the variable region of immunoglobulin genes (IgVs). The mutations are generated by activation induced deaminase (AID) that converts cytosine to uracil in single stranded DNA (ssDNA) generated during transcription. Attempts have been made to correlate SHM with ssDNA using bisulfite to chemically convert cytosines that are accessible in the intact chromatin of mutating B cells. These studies have been complicated by using different definitions of “bisulfite accessible regions” (BARs). Recently, deep-sequencing has provided much larger datasets of such regions but computational methods are needed to enable this analysis. Here we leveraged the deep-sequencing approach with unique molecular identifiers and developed a novel Hidden Markov Model based Bayesian Segmentation algorithm to characterize the ssDNA regions in the IGHV4-34 gene of the human Ramos B cell line. Combining hierarchical clustering and our new Bayesian model, we identified recurrent BARs in certain subregions of both top and bottom strands of this gene. Using this new system, the average size of BARs is about 15 bp. We also identified potential G-quadruplex DNA structures in this gene and found that the BARs co-locate with G-quadruplex structures in the opposite strand. Using various correlation analyses, there is not a direct site-to-site relationship between the bisulfite accessible ssDNA and all sites of SHM but most of the highly AID mutated sites are within 15 bp of a BAR. In summary, we developed a novel platform to study single stranded DNA in chromatin at a base pair resolution that reveals potential relationships among BARs, SHM and G-quadruplexes. This platform could be applied to genome wide studies in the future.

## Author summary

To make protective antibodies against various pathogens, the enzyme activation induced deaminase (AID) introduces mutations into single-stranded DNA (ssDNA) in the variable region of immunoglobulin genes (IGHVs) in B cells, as part of a process called somatic hypermutation (SHM). Here, a bisulfite assay, together with deep sequencing, was used to characterize the accessible ssDNA that represents the substrate of AID in B cells. To deal with issues such as noise in the data, we developed a novel algorithm to more accurately identify bisulfite accessible ssDNA regions (BARs) and applied it to the IGHV4–34 immunoglobulin gene in a human B cell line. Using the new algorithm, we found that location of these BARs recurred in certain subregions of the IGHV4–34 gene. The average size of the BARs is ∼15 bp, which is close to the size of a transcription bubble. We also found that some potential G-quadruplex DNA structures in the IGHV4–34 gene co-located with the BARs but on the opposite DNA strand. Furthermore, we found that, most of the AID induced mutations were near to, but not within, BARs suggesting alternative mechanisms for targeting somatic hypermutation.

## Introduction

High affinity antibodies that can neutralize viruses play a major role in protecting us from viral infections. Such protective antibodies are often generated through the selective somatic hypermutation (SHM) of heavy and light chain antibody variable (V) region genes that encode the antigen binding sites in antibodies. SHM is mediated by the mutagenic enzyme activation induced deaminase (AID) that subjects the V regions to mutation at ∼ 10^−3^/bp/generation which is a million times higher than the frequency of mutation that occurs in other genes [1, 2]. AID is highly expressed during a brief period of B cell differentiation in the germinal centers of secondary lymphoid organs [2]. The substrate for AID is single stranded DNA (ssDNA) [3]. AID induced mutations are largely restricted to the V region exon and to the switch regions that are located downstream and required for isotype switching. This process of AID induced SHM requires a high level of transcription, which is presumably necessary in order to make the ssDNA substrate available [2, 4, 5]. Transcription is a highly regulated process involving changes in DNA structure, DNA binding factors, chromatin structure and the modification of histones and of transcription factors including RNA polymerase II (RNAP II). Furthermore, there is considerable evidence that pausing, elongation and even backtracking and premature termination of RNAP II play important roles in SHM [6–8]. There is increasing evidence that non-B forms of DNA and especially G-quadruplexes (G4) make ssDNA available to directly bind AID and play a role in targeting AID induced mutations to Ig switch regions, thus acting as a key mechanism in class switch recombination, and potentially also in variable regions [9–12].

Since ssDNA is the substrate for AID, it is important to be able to locate and quantify the presence of ssDNA as it is occurring in chromatin in intact B-cells. We therefore developed an assay that would allow us to identify sites of ssDNA with base-pair resolution in native chromatin in intact nuclei in small regions of DNA such as the Ig V region exon [13]. In this assay, nuclei are isolated from crosslinked B cells to stabilize the nucleic acid-protein interactions and treated with sodium bisulfite to convert dC to dU in ssDNA from both top and bottom strands but not in dsDNA. It is important to note that this is completely different from the widely used bisulfite assay for detecting DNA methylation where DNA is first extracted from the chromatin and then this purified DNA is treated with bisulfite to identify methylated and unmethylated bases usually in the promoter where the methylation of DNA often blocks transcription [14, 15]. Furthermore, endogenous DNA methylation is not expected to be a confounding factor here since the rearranged IGHV gene in each B cell is highly expressed [16–19]. In our assay, the DNA that has been modified by added bisulfite while still in the chromatin is extracted and amplified and then sequenced and the uracils that were converted from C to T during amplification are scored as sites of ssDNA that were accessible to bisulfite in the intact nuclei. The technical details are described in the “Materials and methods” section. In our initial studies and in subsequent studies by 3 other laboratories, all of which used Sanger sequencing, it was found that the Ig V regions in primary B cells and in chicken and human B cell lines and off target sites of AID mutation were enriched for tracks or patches of bisulfite accessible dCs compared to other highly transcribed genes that did not undergo AID mutation [7, 13, 20–22].

Transcription was necessary to generate these tracks or patches of bisulfite accessible sites and there was usually no more than one patch of bisulfite accessible DNA per V region and most Vs did not have any bisulfite accessible sites [13, 22]. Like AID mutations, bisulfite accessible sites were found on both strands and the frequency of patches very roughly correlated with the frequency of mutation and the rate of transcription and in one study a gene that mutated at a high frequency also had bisulfite accessible sites [22], but most of the associations between bisulfite accessibility and AID mutations were correlative and applied to whole exons rather than having bp resolution. Most importantly, different laboratories defined bisulfite accessible patches or tracks in different ways. Since the frequency, location and size of the bisulfite accessible site might reveal mechanisms, it is important to decide more rigorously how to define a bisulfite accessible site. In the original work a patch of bisulfite accessible sites was defined as at least 2 [22] or 3 consecutive dCs [13] that had been converted to dT while another study limited the patches to those that had 8 or more nucleotides of which all of the dCs are converted [20]. Two of the studies showed that R-loops were not required for there to be bisulfite accessible regions which is also true of AID induced mutations [20, 22]. With the advent of deep sequencing, it has become possible to look at many more bisulfite accessible regions, providing an opportunity to try to better define a bisulfite accessible site or patch.

Here we have used deep sequencing with unique molecular identifier (UMI) of the V regions in the human germinal center like Ramos Burkitt’s lymphoma B cell line to test for bisulfite accessible regions. This cell line only contains one rearranged heavy chain variable region (IGHV) [23]. We have treated the nuclei of Ramos cells undergoing AID mutation with bisulfite and collected the same population without bisulfite treatment and compared the characteristics of the dCs that underwent C to T conversion. We deep sequenced the heavy chain V regions and used UMIs to minimize the sequencing and background error rate. This is the first time that deep sequencing has been used together with the bisulfite accessibility assay. While we now had the very large amounts of sequence data required to determine the relationship between the bisulfite accessible sites, DNA structure and somatic V region mutation, we needed to develop new analytical tools to analyze all of these data. The tools we have developed uses a novel Bayesian segmentation model that builds upon the concept of a Hidden Markov Model, to determine the characteristics and sites of accessible ssDNA in chromatin and to compare them to the sites of AID induced mutation and to non-B-DNA structures such as G-quadruplexes.

## Results

### Deep sequencing of libraries prepared from bisulfite treated nuclei

**Fig 1:**
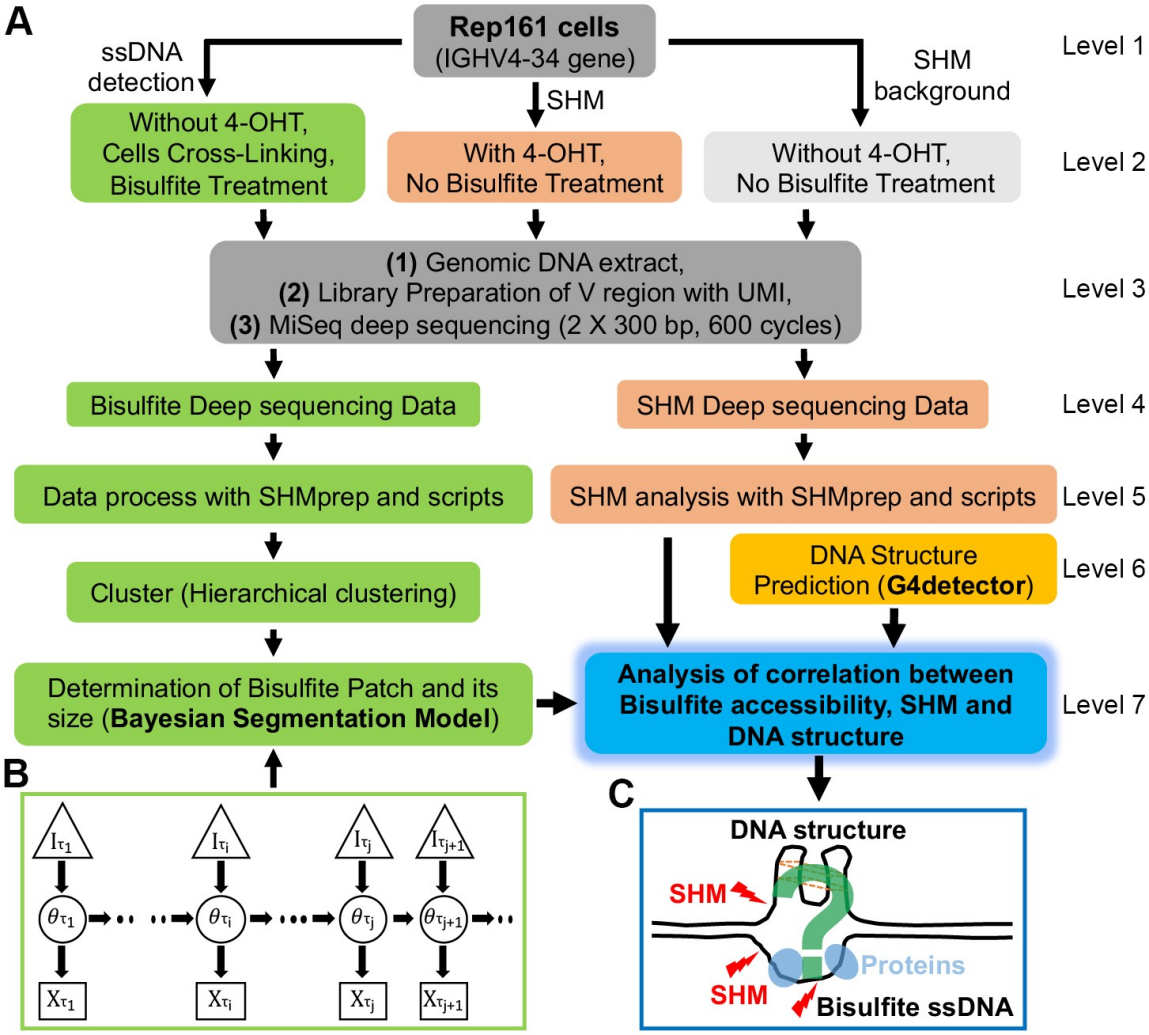
Pipeline and schema of the novel Bayesian segmentation model developed for this study. Overall, this study includes 5 parts: (A, level 1–3), Separate preparation of libraries with UMI for both bisulfite accessible ssDNA and SHM and deep sequencing using Illumina MiSeq. (A, left part of level 4–7 in green background and panel B), Using the Bayesian segmentation model to determine the BARs; (A, right part of level 4–5), Calculate SHM from the same cell line; (A, right part of level 6), Predicting G-quadruplex structures for IGHV4–34 gene; (A, right part of level 7 and panel C) Studying the spatial correlation between BARs and G-quadruplex to analyze the relationship between BARs and SHM at a base-pair resolution. A detailed explanation for this Bayesian segmentation model can be found in the main text (Results). In panel C, red lightning marks represent SHM by AID and panel C is a cartoon illustrating the possible correlation among SHM, BARs and the DNA structure. ssDNA: single stranded DNA. SHM: somatic hypermutation. 4-OHT: 4-Hydroxytamoxifen. UMI: unique molecular identifier.

We used UMI (unique molecular identifier) based deep sequencing to examine the bisulfite accessibility of the IGHV region in the chromatin of human B cells using a variant of the Ramos B cell line that can be induced to undergo V region hypermutation. In this Ramos Rep161 reporter system [7, 24], the mutations in the endogenous IGHV4–34 gene can be readily induced by treating with 4-Hydroxytamoxifen (4-OHT) that drives AID from the cytoplasm into the nucleus (Fig 1A, level 2). For the bisulfite experiment, the cells were not treated with 4-OHT to avoid the complication of distinguishing AID and bisulfite induced C to T mutations (Fig 1A, green box on level 2). As described in the “Materials and methods” section, the cells were cross-linked with formaldehyde and nuclei were prepared and treated with bisulfite to convert the accessible Cs to Us in both top and bottom strands [13] (Fig 1A, level 2). After extracting the genomic DNA, the V regions were amplified by PCR using specific primers with UMI [25]. During the PCR process, all of the Us should be replicated to Ts [26] and identified using paired-end (2 × 300 bp) deep sequencing on the Illumina MiSeq platform (Fig 1A, level 3).

After sequencing, the raw data were processed using the SHMprep program (http://www.ams.sunysb.edu/∼maccarth/software.html) with a filter applied for including only consensus sequences constructed from ≥ 3 identical UMIs (Fig 1A, green box of level 5). Any sequences containing indels were also removed from further analysis. A total of 147,293 high quality unique sequences were collected based on the UMI. The clean assembled data were processed to extract the bisulfite conversion and to generate a mutation matrix (sequence × position—S1 to S3 Datasets) for downstream analysis.

### Bisulfite accessible regions were identified using a Bayesian segmentation model

Previous papers defined a Bisulfite Accessible Region (BAR) by measuring the length of the track or patch of nucleotides in which consecutive runs of Cs were converted to T [20–22]. However, scoring only regions in which a certain number of consecutive Cs have all been converted to Us does not account for several important possibilities. By definition, single converted Cs were previously ignored even if they were recurrent in many B cells. Thus, a patch of ssDNA in regions with a low abundance of Cs might be missed. In addition, a larger patch in which a single internal C is sometimes occupied by DNA binding proteins, such as AID itself, would be scored as two smaller patches on either side of the unconverted C. It was also unclear how to score the size of patches where the underlying sequence did not have one or more closely linked Cs at its 3’ or 5’ edge. Furthermore, under the assumption that there exist recurrent BARs, minor differences due to DNA or protein movements will lead to noise in ssDNA exposure at patch edges. Thus, there is a need for a statistical method suitable for high-throughput data that addresses the above issues to provide robust BAR profiles.

In order to begin to examine some of these possibilities, DNA was examined from reporter (Rep161) Ramos cells whose cross-linked nuclei had been treated with bisulfite in situ (see “Materials and methods” section). The bisulfite mutation matrix was separated into top strand and bottom strand based on C>T and G>A mutations respectively. The dataset for each strand was clustered using hierarchical clustering (see “Materials and methods” section) into 6 groups based on sequence similarity (Fig 1A, green box of level 6). To analyze the bisulfite converted sequences, we need to detect the changes of bisulfite accessibility. In previous studies dealing with DNA copy number variation [27] and Hi-C data [28], hypothesis tests were used for change detection. But instead of dealing with abrupt changes, our data indicated a need to capture continuous changes. So, a novel Bayesian model was developed to characterize the BARs (Fig 1A, green box of level 7). Hidden Markov Models (HMMs) are usually used to estimate signals in sequence data in which signals switch among different states (accessible or non-accessible). HMMs usually deal with problems having a finite number of discrete states, but response rates in our sample can change even within a single accessible segment and hence have continuous states. Our Bayesian segmentation model is an extension of finite-state HMMs to continuous states [29]. Bayesian models are suited to describing uncertainty, and in our case, this is primarily the uncertainty about the change of response rate (to bisulfite) among accessible and non-accessible regions. Two previous studies had approached genomic or protein segmentation problems with finite-state Bayesian models [30, 31]. In our model, the accessibility parameter has a continuous state and hence can change to any real value. Because in our data it appeared that accessibility could change to any continuous valued state, it suggested that a continuous state hidden Markov model would be more appropriate. In addition to this, the Bayesian model we developed can also provide accurate analytic posterior estimations by means of a dynamic programming algorithm.

A schematic description of our model is shown in Fig 1B, across three layers of variables, one observed and two latent (the latter will be estimated). The arrows represent dependence relationships. The model can be considered to move between discrete sites represented by the subscripts *τ*_*i*_, each representing a successive C nucleotide. The bottom layer ($X_{\tau_{i}}$) corresponds to the observed mutation frequency at that site, defined as the number of mutations divided by total reads. The middle layer are latent variables ($\theta_{\tau_{i}}$) and correspond to the probability that the particular C site is accessible. The upper layer (latent) variables, $I_{\tau_{i}}$, are indicator variables (can take a value of 0 or 1), where a 1 corresponds to site where the accessibility probability ($\theta_{\tau_{i}}$) changes, which includes boundaries of a BAR or quantitative changes within it. Thus, for example, if the BAR starts at position *τ*_*i*_, so $I_{\tau_{i}}=1$ and $\theta_{\tau_{i}}$ has a new value that is higher than the *θ* value(s) immediately before it. Furthermore, this change should be associated with a large $X_{\tau_{i}}$. The subsequent $I_{\tau_{i+1}}\ldots I_{\tau_{j}}$ values within the BAR could all be 0, which in turn means that the $\theta_{\tau_{i+1}}\ldots \theta_{\tau_{j}}$ all remain unchanged at $\theta_{\tau_{i}}$ and the corresponding response should also remain high. Assuming *τ*_*j*_ is the last site within the BAR, then $I_{\tau_{j+1}}=1$, so $\theta_{\tau_{j+1}}$ becomes small again and the corresponding observed $X_{\tau_{j+1}}$ from the data should also be small. Sometimes, at the boundary of a BAR, there could be two or more consecutive *I*_*τ*_ equal to 1, describing a smoother change. In our model, each $I_{\tau_{k}}$ is a Bernoulli random variable, whose probability to be 1 can be estimated. Each $\theta_{\tau_{k}}$ comes from a Beta prior distribution if the corresponding $I_{\tau_{k}}$ is 1. The posterior value of each $\theta_{\tau_{k}}$ can also be estimated. The algorithm for estimating the model parameters from the data is described in the “Materials and methods” section.

**Fig 2:**
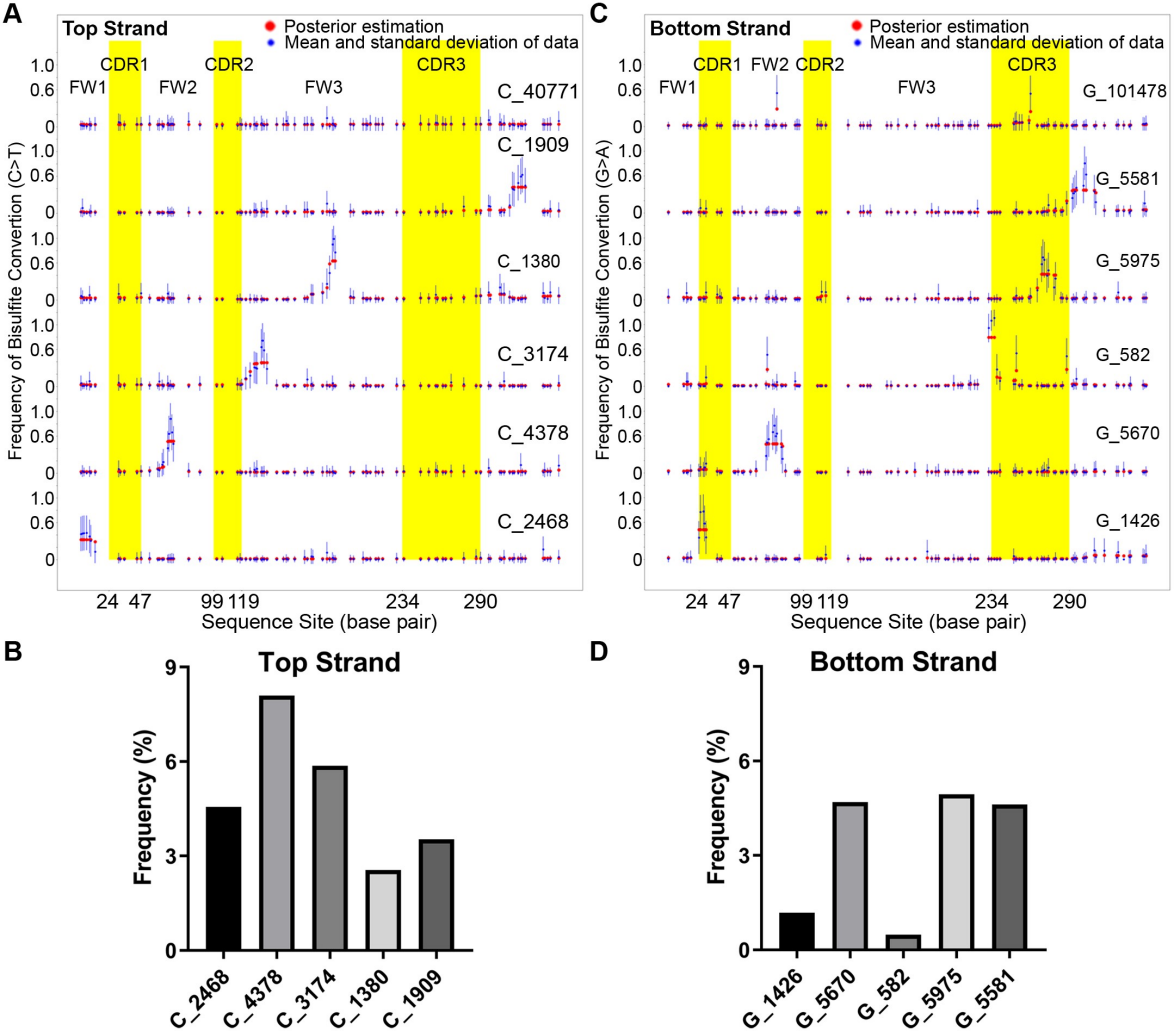
Distribution of bisulfite accessible regions in IGHV4–34 of Ramos analyzed by Bayesian segmentation model. (A) Distribution of the bisulfite accessible regions in the top strand as indicated by C>T mutations. Sequences were clustered by hierarchical clustering and then the patch boundary in each cluster was determined by the Bayesian Segmentation Algorithm. Blue dots with error bars represent the frequency of converted C in each nucleotide position and the red dots indicate the probability of having a converted C using the Bayesian Segmentation Algorithm. The number of sequences in each cluster is listed at the right side of the corresponding cluster. The first cluster (at the top) does not have a typical patch region. The X axis is the nucleotide position within the IGHV4–34 sequence in Ramos. The regions in yellow color background are CDR1, CDR2, and CDR3 from left to right. (B) The frequency of cluster with bisulfite accessible region. For each cluster, the frequency was calculated as the ratio of sequences in the cluster among all sequences. (C) Distribution of bisulfite accessible regions in bottom strand determined by G>A mutation. (D) Frequency of cluster with bisulfite accessible region in bottom strand. The order of the columns corresponds to the clusters, from bottom to top, of panel B.

The results of combining the hierarchical clustering and the Bayesian segmentation model, as applied to our data, are shown in Fig 2. The CDRs are the regions that encode the part of the antibody variable region protein that creates the antigen binding site and the FWs are the regions that stabilize the CDRs to create the antigen-binding site [32]. The deep sequencing started near the 3’ end of FW1 so that we could extend through CDR3. The majority of the Ramos IGHV4–34 V regions did not have any statistically significant clusters in the top strand (Fig 2A top row, C_40771, 75.4%) as described by the C>T mutations. However, 24.6% of the sequences could be separated into 5 clusters, each containing a single major BAR, in different locations of the V region. Each cluster is labeled by the strand and the number of unique sequences so, for example, “C_1909” is a top strand cluster containing 1909 unique sequences from a total of 54,080 sequences considered. Starting at the bottom row of Fig 2A and moving upwards row by row, these 5 distinct accessible regions were respectively distributed in framework FW1 close to complementary determining region CDR1, in the middle area of FW2, in the 5’ region of FW3 near the CDR2, in the middle of FW3, and in the region just 3’ to CDR3. As shown in Fig 2B, the percentage of V regions with clusters of BARs in FW2 (8.1% of sequences) is the highest, followed by the V regions with the BAR near CDR2 (5.9%), suggesting that FW2 and the subregion 5’ to FW3 are more accessible compared with other sub-regions in the top strand of the V region.

For the bottom strand, as displayed in Fig 2C, 15.93% (19,234 out of 120,712) of the sequences were found to have one of 5 clusters (rows 2–6 of Fig 2C) containing a nontrivial BAR based on the Bayesian algorithm. Interestingly, for the bottom strand, the accessible regions mainly locate in the CDR1 (G_1426, 1.18%), FW2 (G_5670, 4.7%) and two BARs in/near the CDR3 (G_5975, 5.0% and G_5581, 4.6%). The FW2 and CDR3 subregions are the most accessible regions, as measured by clone frequency, in the bottom strand (Fig 2D). The frequency of bisulfite accessibility in the bottom strand (15.93%) is significantly lower than the top strand (24.6%, chi-squared *p* < 2.2 × *e*^−16^). In the top strand, the FW2 BAR is also the most accessible region (Fig 2B) contributing most of the difference between the two strands. Many of the top strand BARs do not return to baseline and have intermediate values at their 3’ ends probably because there are no Cs that can be converted to U at those borders. In addition, especially in the bottom strand, there are some single Cs that are recurrently targeted by bisulfite in the many V regions that do not have patches (G_101487, top row of Fig 2C) and elsewhere such as in CDR3 in G_582, which is a subgroup with a few single site patches and one larger one in CDR3. In summary, using our newly developed algorithm, we found the BARs do not seem to randomly distribute on the V region suggesting that they may have some functional significance.

### The size of the BARs is different in different subregions of the Ramos V region

**Fig 3:**
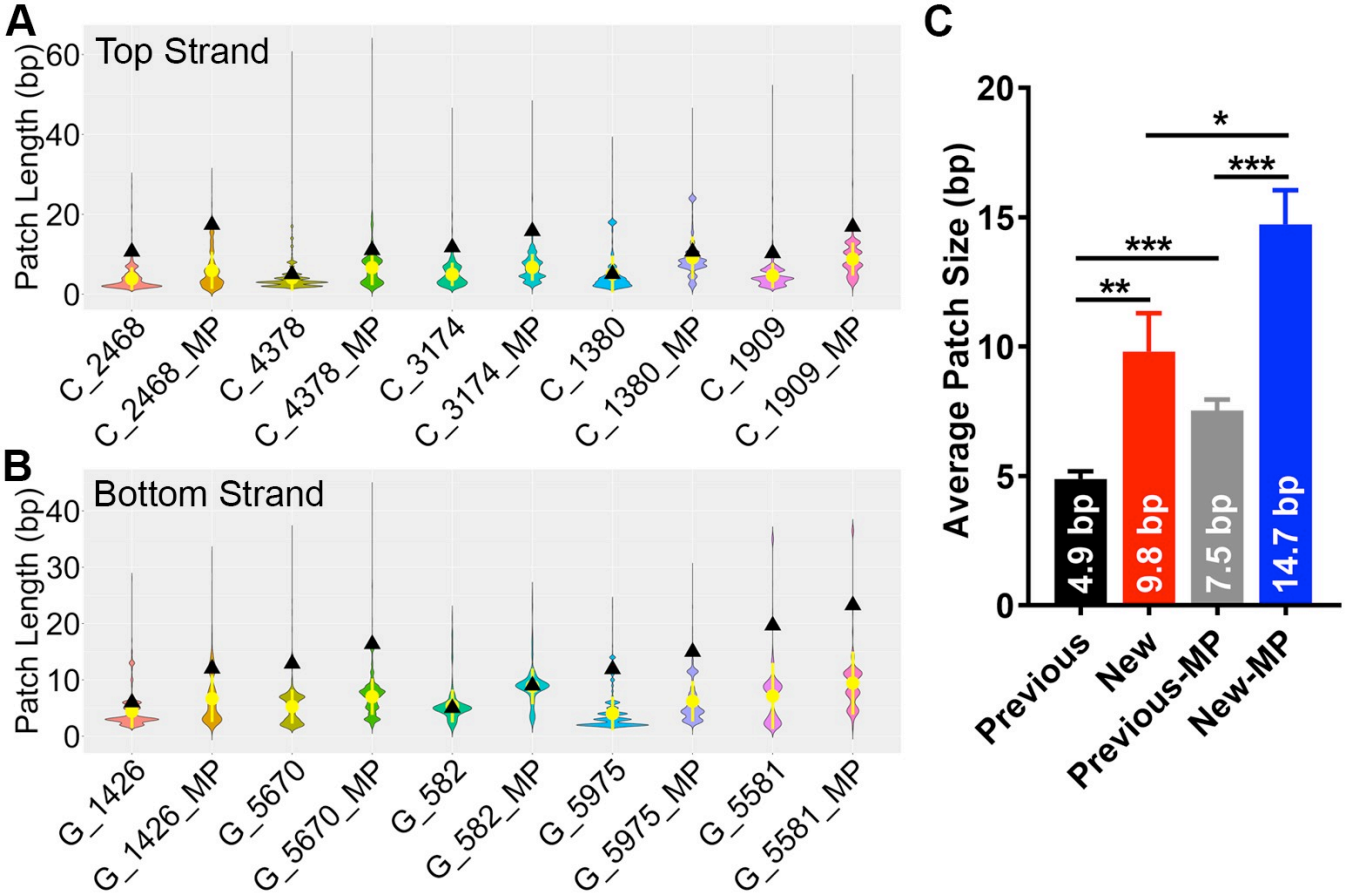
Comparison of length distribution of BAR in each cluster calculated by two different methods. For each cluster, on the one hand, the size of bisulfite patch was calculated as the distance between two or more consecutive converted Cs as reported in previous papers [22], on the other hand, the length of bisulfite accessible region was calculated based on the Bayesian Segmentation Algorithm to find the boundary of potential accessible regions. (A) The patch size of bisulfite accessible region for each cluster in top strand. Y axis represents the patch length and the X axis indicates each cluster. The black triangles show the patch size (bp) calculated by the Bayesian model developed in this study, while the violin plots with mean value (yellow dot) are the patch length determined by previous method. In the X axis, from left to right, the name of each cluster corresponds to the cluster in Fig 2A ordered from bottom to top. The cluster name with postfix “_MP” means the patch is redefined to calculate the distance between two midpoints flanking the consecutively converted Cs, i.e. the midpoint between the terminal converted C and its nearest un-converted C. (B) The patch size of bisulfite accessible region for each cluster in bottom strand. (C) the average size of patches that was calculated by different definitions as shown on X-axis. Previous: definition used in published papers [22]. New: the Bayesian Segmentation model developed in this study. Previous-MP: previous definition with the mid-point concept proposed here. New-MP: Bayesian model with mid-point concept. For each group, the patch size from both top and strands were included. Error bars represent Standard Error of the Mean. *: *p* ≤ 0.05, **: *p* ≤ 0.01, ***: *p* ≤ 0.001. P values were calculated using an unpaired Student’s T-test in Graphpad Prism 8 software.

Using our method we were able to estimate the average patch size for each of the clusters that contained BARs, as shown by the black triangles in Fig 3. We found that BAR sizes vary between the different gene sub-regions, ranging from 5 bp to 20 bp for both top and bottom strands. In the top strand (Fig 3A), the clusters in the middle of FW2 (C_4378) and FW3 (C_1380) contain smaller patches (∼5 bp), while BARs in the 3’ part of FW1 region which is near CDR1 (C_2468), 5’ of FW3 which is near the CDR2 (C_3174) and the region past the CDR3 (C_1909) are bigger and range from 10 bp to 12 bp in size. In the bottom strand (Fig 3B), in the junction regions between FW1 and CDR1 (G_1426) and between FW3 and CDR3 (G_582), the patch sizes are relatively small, at 6 bp and 5 bp respectively, whereas in FW2 (G_5670) and CDR3 (G_5975 and G_5581), the BAR lengths are relatively large, ranging from 12 bp to 20 bp in size.

In previous papers, BARs were defined as a patch in which 2 or more consecutive Cs had been converted to Us within each individual sequence and with the BAR ending at the last converted C on either end [20, 22]. For comparative purposes, we calculated the patch size distributions using this previous definition. The results are shown as violin plots in Fig 3A and 3B, with the mean and standard deviation shown in yellow. As shown in Fig 3C, the average size of BARs using the previous calculation (average size: 4.9 bp) is significantly smaller than the average size of BARs calculated using our model (9.8 bp, *p* < 0.01), with the majority of BARs being between 2 bp and 5 bp and the average patch size ranging from 3bp to 5bp (yellow dot) for all subregions for both top and bottom strands.

However, in the above result, both our new algorithm and the previous method only considered the distance between the two consecutive Cs and assumed that regions which do not contain Cs flanking outside of the terminal converted Cs are not accessible. Assuming that the BAR ends exactly with the last converted C leads to a conservative measure of BAR size because the actual BAR boundary could in fact be anywhere between the last converted C and the next unconverted C (the assay cannot observe accessibility for any non-C sites in between). Because in our model we assume BAR boundaries occur along DNA as a Poisson process so, given that there exists a boundary between two consecutive Cs, the expected location of the boundary should be at the midpoint of the two Cs. Thus, we introduced a new definition for the patch, extending it to the two midpoints on either side. When applying this definition to both the previous and our novel methods, the patch size is significantly increased (Fig 3, Previous: 4.9 bp vs Previous-MP: 7.5 bp, *p* < 0.001; New: 9.8 bp vs New-MP: 14.7 bp, *p* < 0.05). For the previous method, the patch size ranges from 5 bp to 10 bp (Fig 3A and 3B) with average size being 7.5 bp (“Previous-MP” in Fig 3C), while for our new model, it ranges from 9 bp to 24 bp (Fig 3A and 3B) with average size being 14.7 bp (“New-MP” in Fig 3C) that is significantly larger than the “Previous-MP” (7.5 bp, *p* < 0.001). By this new definition, our new method probably can include the possible accessible regions that do not contain Cs and flank outside of the terminal accessible Cs. Interestingly, we observed the average size of BARs using “New-MP” method (14.7 bp) are similar in length to estimates of the transcription bubble at 12–14 nt [33–35], which has in many studies of SHM been considered a potential target for AID (and plausible BAR) given it involves single stranded DNA particularly on the coding, or non-template, strand that is not occupied by polymerase.

### Predicted G-quadruplex (G4) structures co-locate with BARs on the opposite strand

An obvious question is why the BARs are only located in particular sub-regions of the IGHV4–34 gene and not randomly distributed across the whole gene (Fig 1C). We speculated that DNA secondary structure might play a role since previous papers had shown that the G4 structures play important roles in class switch recombination by regulating and/or interacting with AID [10] and a mutant form of AID that is unable to bind G4s had a significantly reduced capacity to generate both CSR and SHM [36]. More generally, G4s also play an important role in regulating transcription pausing near transcription start sites [37, 38].

We used G4detector (https://github.com/OrensteinLab/G4detector)—a deep learning-based program that uses a convolutional neural network—to estimate the potential of G-quadruplexes forming on the top and bottom strands throughout the Ramos IGHV4–34 gene (Fig 1A, golden box on level 6). G4detector was originally trained on a combination of data from in vivo ChIP-Seq and in vitro G4-seq, a high-throughput biochemical assay that quantifies G4 probabilities genome wide via modified deep sequencing in the presence of G4-promoting agents [39, 40]. G4detector generates a single estimated probability that the input sequence will form a G4, and greatly outperforms comparable methods such as Quadron [41] and G4Hunter [42] in predicting these probabilities [39]. However, a limitation of G4detector is that it does not suggest which parts of the input sequence may have formed the G4 structure and contributed to the final estimate. In order to assess the regions that may be engaged in forming the G-quadruplex itself, we used the Integrated Gradients method (https://arxiv.org/abs/1611.02639) to measure the relative contribution of each individual site in the input sequence, to the predicted output (see “Materials and methods” section).

**Fig 4:**
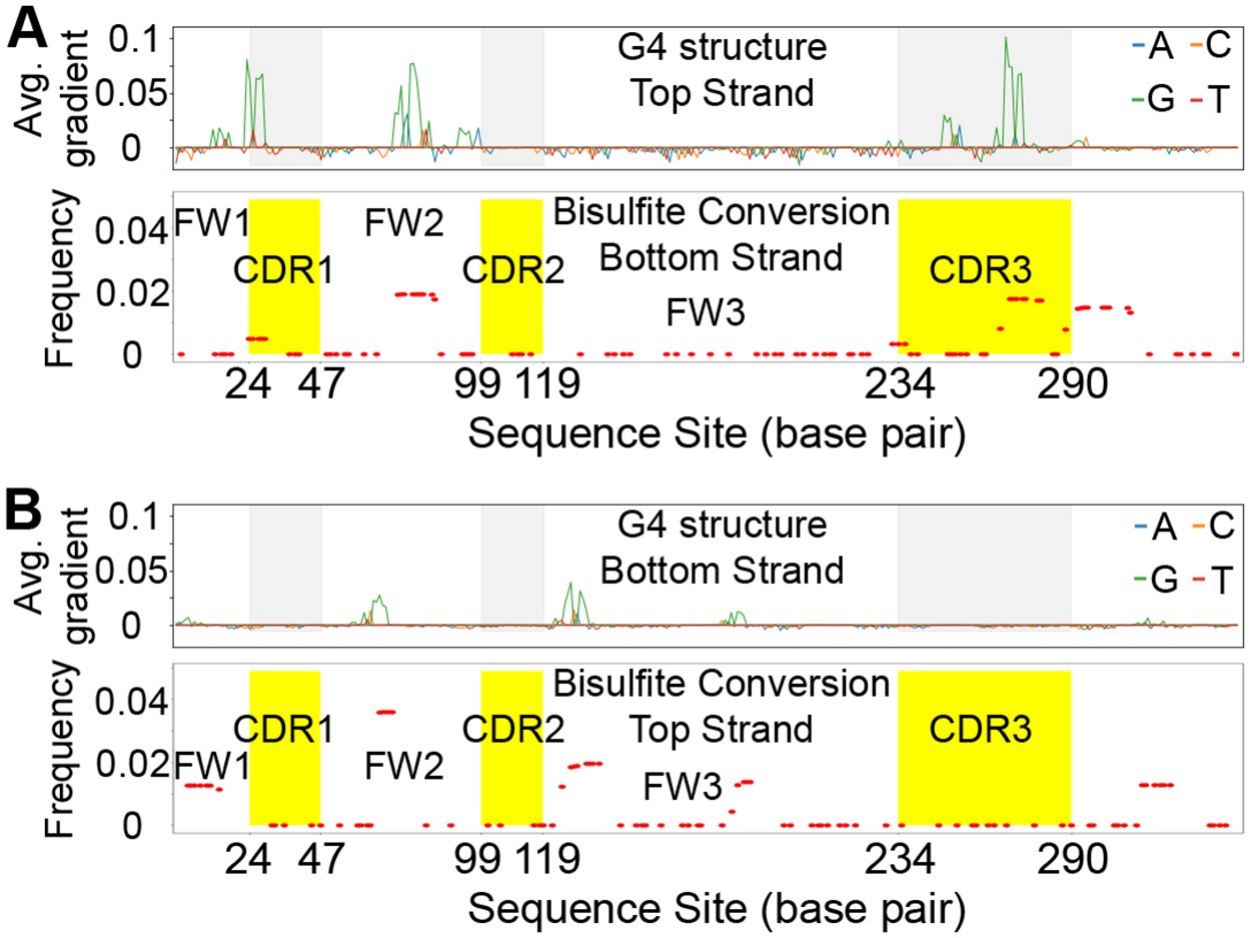
The potential spatial correlation between BAR and DNA G-quadruplex structure in IGHV4–34 gene in Ramos. (A) The sites having potential G4 structure in the top strand colocalize with the sites having BARs in its corresponding bottom strand. Top panel shows the position of the predicted G4 structures in top strand. X-axis is the nucleotide position of IGHV4–34 gene and Y-axis represents the average gradient of the predicted G4 structure in each site. The regions with gray background are CDR1, CDR2 and CDR3 from left to right. Different colors represent individual bases that contribute to the predicted G4 potential. The bottom panel shows all the BARs from different clusters together. Y axis represents the frequency of BARs in each position. The regions with yellow background are CDR1, CDR2 and CDR3 from left to right. (B) The correlation between predicted G4 structure on bottom strand and the BARs in its corresponding top strand.

Because G4detector only accepts input sequences of length 297 nt, and the V region in Ramos is of length 346 bp, we used a moving window to evaluate G4 potential along the entire gene. Thus, applying G4detector to the Ramos top strand sequence, we found a mean G4 probability of 49.5% (range 26.4% to 73.3%). Integrated Gradients revealed multiple G-repeats that may be involved in creating one or more G4s structures (Fig 4A and 4B for top and bottom strands, respectively). The top strand contained several areas with high contributions, mainly localized to CDR1, FW2, and CDR3, and to an extent, FW1 (Fig 4A). When mapping these areas to BARs (for all data aggregated) in the top strand, there was no overlap between these two regions (not shown). However, we did observe a significant association of highly contributing G-repeats with bottom strand BARs (*R* = 0.57, *p* = 2.9 × *e*^−10^, Fig 4A) so the G4 structures in the top strand are compared to the BARs in the bottom strand in Fig 4A which did reveal a relationship.

We then repeated this process to assess G4 potential in the bottom strand of the Ramos V region. Here, G4detector predicted the bottom strand to have an average G4 probability of 11.2% (range 8.4% to 15.0%). Integrated Gradients of the bottom strand sequence revealed multiple areas, albeit with lower contribution scores than the top strand, which is consistent given its lower G4 potential. Overall, we observed one G-repeat found in FW2, as well as several G-repeats in FW3 (Fig 4B). In addition, we observed these areas significantly intersected with BARs in the top strand, not with the bottom strand (*R* = 0.8, *p* = 4.5 × *e*^−21^, Fig 4B). Taken together, these data suggest that BARs can co-locate with G-quadruplexes on the opposite strand.

### Analysis of somatic hypermutation pattern of the Ramos V region by deep sequencing

Previous studies in a variety of B cell systems had shown that the frequency of ssDNA in whole V regions as determined by bisulfite assay positively correlates with transcription and also with the frequency of AID induced SHM within the same genetic regions in both the Ig locus and AID off-target genes [21, 22]. This was consistent with the fact that biochemically ssDNA is the substrate for AID [3, 43]. However, since there were no studies that used deep sequencing to provide sufficient data to explore the relationship of ssDNA and AID induced somatic mutation at high resolution, there was not clear picture of whether the SHM sites are in or near the bisulfite accessible ssDNA.

In order to study the spatial correlation between BARs and AID induced V region somatic hypermutation in Ramos (Fig 1C), the same reporter cell line that had been examined for BARs was treated with 4-OHT for 7 days to drive AID into the nucleus and cause somatic mutations in the V region (Fig 1A). Based on previous studies [21, 22], we expected that the frequency of AID induced mutations would be much lower than the frequency of bisulfite induced mutations. V region amplicon libraries were prepared from genomic DNA with UMIs for deep sequencing (Fig 1A, orange boxes and level 3). The raw data was again processed by SHMprep with the same parameters used for the bisulfite dataset (Fig 1A, orange box of level 5, S4 to S9 Datasets). In total, 262,956 unique high-quality sequences were collected and a new mutation matrix was generated. The frequency of mutation at C is 0.085% in both the top and bottom strand in this SHM dataset. This is in contrast to the bisulfite dataset in which the rate of bisulfite conversion of C in top strand is 0.73% and 0.75% for the bottom strand. We also sequenced the IGHV4–34 region from reporter cells that were treated with neither 4-OHT nor bisulfite (Fig 1A, right box of level 2). The background mutation rate of C to T is 0.05% in top strand and is 0.04% in the bottom strand. The background mutation is probably due to both the leakage of AID-ER into nucleus and the undetectable level of the endogenous AID. In summary, the frequency of C mutation by bisulfite is much higher than the 4-OHT induced mutation and the background mutation frequency. This was expected since even though AID has a relatively high mutation rate in Ramos cells of ∼ 10^−4^/bp/generation, this is much lower than bisulfite which will convert C to T in any accessible C.

As already noted, the IGHV gene in this reporter cell line is IGHV4–34*01. To further confirm that AID induced mutations by 4-OHT treatment in this reporter cell line are representative of the normal pattern of SHM of IGHV4–34*01 genes in human primary B cells, we compared the distribution pattern of SHM from this induced cell line with the SHM pattern from a previously published deep-sequencing dataset of IGHV4–34*01 in human primary B cells that had not been analyzed site by site [44]. The SHM pattern in our reporter cell line is very similar to the pattern in the human primary B cells (S1(A) and S1(B) Fig, *R* = 0.84, *p* < 2.2 × *e*^−16^). In particular, IGHV4–34 does not have a high frequency of SHM in CDR2 either in vivo or in Ramos cells, even though CDR2 is often highly mutated in many other human V regions. This is probably due to the intrinsic characteristics of IGHV4–34*01 [44, 45]. These data together with the previous study using IGHV3–23*01 in Ramos showing a high correlation between the Ramos cell line and a human database of IGHV3–23*01 mutations [46] shows that the pattern of SHM in this 4-OHT inducible Ramos cell line quite accurately reflects the SHM process of human primary B cells.

### The SHM of highly mutated sites correlates not only with the bisulfite frequency of the corresponding single site but also with their distance to the BARs

**Fig 5:**
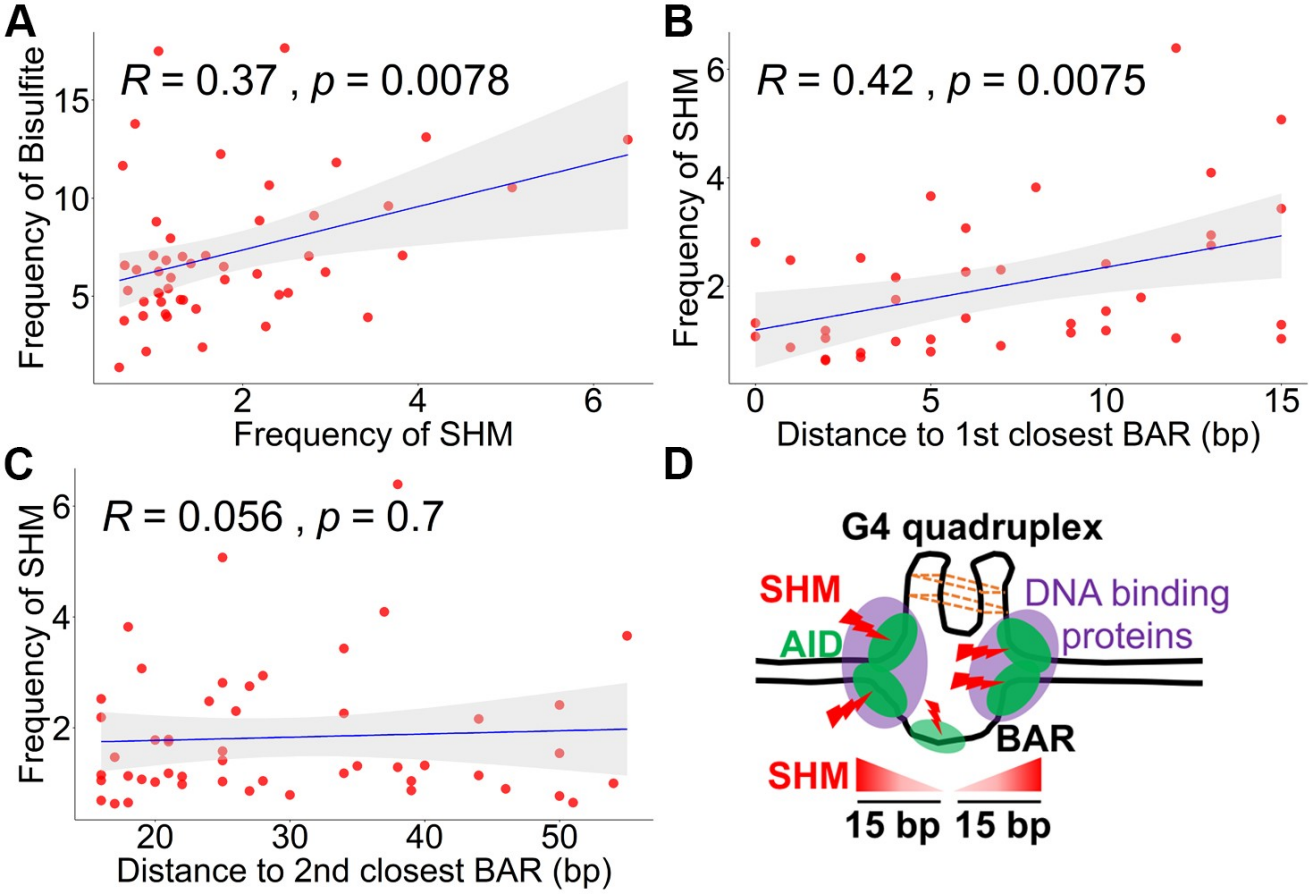
Spatial correlation between the highly mutated sites in SHM and the BARs. (A) the relationship between the frequency of SHM for the top 25 highly mutated sites from both strands (50 sites in total) and the corresponding frequencies of bisulfite accessibility. (B) the correlation between the same 25 highly mutated sites from both strands and their respective distance to the closest BARs with 15 bp context. (C) the correlation between the same 25 highly mutated sites from both strands and their respective distance to the second closest BARs, all of which are beyond 15 bp. The frequency of Bisulfite and frequency of SHM shown in panel A-C are in the form of 10^−3^ for better visualization. (D) The potential model displaying the correlation among SHM, BARs and G4 quadruplex. The red lightning symbol shows SHM introduced by AID (green oval). The purple ovals represent protein complexes that occupy the ssDNA region to facilitate AID to mutate. The bottom triangles with gradient red color shows SHM which deeper red color represents higher mutation within 15 bp context.

In order to correlate the AID mutations with the Bisulfite analysis (Fig 1C), we separated the AID mutation dataset into top and bottom strands, based on the C>T and G>A (C on the bottom strand) mutations. S2(A) Fig shows the two datasets together (bisulfite accessibility above, SHM frequency below), with the vertical bars colored according to strand. As noted, AID induced SHM occurs at a much lower frequency than the mutations introduced by bisulfite. We first evaluated whether there was a direct correlation between bisulfite accessibility and SHM site-to-site for all C or G sites and found there was no significant correlation on either the top (*R* = −0.042, *p* = 0.69, S2(B) Fig) or bottom (*R* = −0.02, *p* = 0.84, S2(C) Fig strand. Because in the analysis in the previous section where we found an association between predicted G4s and BARs on the opposite strand, we compared bisulfite accessibility with SHM frequencies on the opposite strand. A direct site-by-site comparison between C and G sites (C on the opposite stand) is not possible, so we compared the two strands using Gaussian kernel smoothing (*σ* = 1). Again, we did not find a significant correlation (top SHM vs bottom bisulfite: *R* = 0.032, *p* = 0.76; bottom SHM vs top bisulfite: *R* = −0.02, *p* = 0.84, S2(D) and S2(E) Fig). However, there are clearly some single sites such as residues 53, 86, 179 and 197 (indicated with arrows in S2(A) Fig) where there are highly recurrent and coincident AID and bisulfite mutations on both strands. Based on this we determined if there is a correlation between sites with the most frequent AID mutations and bisulfite frequency for both strands. We chose the set of sites with the most AID mutations, testing a range for the number of sites chosen. For example, Fig 5A shows the results for the 25 most AID mutated sites on both strands (50 sites), where we found a modest correlation that is however statistically significant (*R* = 0.37, *p* = 0.0078). Similar results are found if we choose the 20 (*R* = 0.37, *p* = 0.019), 30 (*R* = 0.35, *p* = 0.0062) or 40 (*R* = 0.26, *p* = 0.019) most highly mutated sites for both strands. Because noise due to sampling is expected to be higher for sites with lower mutation frequencies, and because there are many more such sites, this may explain why we do not observe a significant correlation when all sites are considered.

Previous studies have shown that although AID induced SHM correlates with the position and sequence context in IGHVs [44, 45, 47], it is not known how this relates to the local ssDNA accessibility to AID. However, from the above distribution analysis of BARs, we found the BARs seem to be located in particular sub-regions of this IGHV gene rather than being randomly distributed. Also, the sites with relatively high AID induced SHM appear to be more accessible to bisulfite (Fig 5A). Therefore, we speculated that the BARs may provide a locally accessible context that facilitates the initiation of AID mutation on a single accessible C. To take advantage of our new algorithm, we further compared the BARs we extracted from each cluster (Fig 2) to the SHM data. Since the top 25 highly mutated sites from both strands gave us slightly higher R and lower p values for the correlation between SHM and the respective bisulfite frequency site to site, for the SHM data, we chose these same 50 sites (25 from each strand) and computed pairwise distances to each of the 5 BARs we found on each strand (Fig 2). Consistent with the importance of sequence context in SHM [44, 45, 47], most of those 50 sites are located in AID preferred motifs like W**GC**W (W = A/T), WR**C** (R = A/G) or **G**YW (Y = C/T) (S3 Fig, mutation sites are bolded in motifs).

We next analyzed the correlation between the frequency of SHM in each site of the top 50 highest mutated C and G sites and their distance to the BARs, considering distances of up to 15 bp since this is the approximate size of a transcription bubble [33–35] (a plausible BAR mechanism) and also approximately the average size of BARs (14.7 bp) calculated using the “mid-point” method (Fig 3C). We found that SHM frequency is *positively* correlated with their distance to the closest BARs within this 15 bp context (*R* = 0.42, *p* = 0.0075, Fig 5B), suggesting there may be an optimal distance for which the BAR can influence a highly mutated site. As a negative counterexample, we found no correlation between the SHM frequency and their distance to the second closest BARs which are all >15 bp away (*R* = 0.056, *p* = 0.7, Fig 5C). We conclude that, while some BARs do co-locate with or are close to highly mutated SHM sites, the frequency of SHM of these sites seems to be increased when they are slightly separated but within 15 bp of a BAR. Future experiments using site directed mutagenesis of the V region should reveal if there is a causal relationship between BARs and SHM at those nearby locations.

## Discussion

Mutational targeting within the Ig genes is not uniform and can only be partly explained by the increased density of AID and Pol *η* hotspots and the sequence context, for example, within the CDRs in some V regions [44–46]. Given that the primary substrate for AID is ssDNA, understanding where and why ssDNA is made accessible may reveal why there are preferred subregions for mutational targeting and provide new insights into underlying molecular and biochemical mechanisms of V region hypermutation. Several previous studies have assessed ssDNA accessibility directly using a bisulfite-based assay that deaminates exposed Cs in ssDNA but not dsDNA in the native chromatin environment, followed by Sanger sequencing. Previous studies using Ramos cells and other cell lines suggested that accessibility was due to DNA supercoiling that occurs in the wake of the transcription bubble [6, 22, 48, 49]. They also showed that this applied to off-target sites that are also targeted by AID, albeit at a much lower rate. In this and other studies that have used this assay, a bisulfite accessible patch has been defined as two or more consecutive deaminated Cs. This definition was justified by the finding that there was a significant difference in the frequency of such patches between the V region and the downstream constant (C) region and some other highly transcribed genes which do not undergo AID mutation [13]. Using the less conservative definition of a single converted C, the difference between V and C was not significant and these sites were therefore ignored. However, the underlying reason that C regions do not mutate is not known but could well be due to other reasons than lack of accessibility.

Deep sequencing of IGHV genes is now commonly used to characterize SHM, and a variety of software tools with many features are available to process these data such as pRESTO [50], Change-O [51], partis [52] and VDJServer [53]. More recently, high-throughput techniques that use UMIs to reduce sequencing error to levels below that of Sanger sequencing have become available [54]. UMI-based sequencing has allowed us to generate tens of 1000s (rather than 100s) of unique V region sequences without many rounds of PCR duplication [55, 56]. However, processing such large datasets creates new challenges. Given such a dataset from bisulfite-treated V regions, we expected to be able to robustly identify recurring sites of bisulfite accessible regions and better define their characteristics. Since AID mutations occur with similar frequencies on both strands of DNA, we made the assumption that recurring ssDNA patches should exist on both DNA strands but that these are affected by various sources of noise including transient protein binding to the ssDNA (including AID itself) and DNA breathing. Due to the presence of noise and the large amounts of data involved, it was necessary to develop a statistical method that could estimate the positions and sizes of the BARs even in the presence of noise. The method we developed starts with a hierarchical clustering step that broadly separates the sequences into groups having similar patterns of bisulfite accessibility. A subsequent step estimates the patch positions and sizes for each cluster using a Bayesian method that also measures confidence for each estimate. The method is available as a separate Python script with detailed instructions which can be used to study BARs genome wide or in particular gene loci. This could be useful since patches of ssDNA may mark a variety of cellular processes like transcription pausing, elongation and backtracking. This should also allow investigators to use inhibitors or genetically defective cells or animals to study the contribution of transcription or other processes to the BARs in specific loci.

The hierarchical clustering step showed that most of the endogenous IGHV4–34*01 regions in the Ramos human B cell line contained either no bisulfite accessible sites or a single bisulfite accessible base pair. Because, as noted above, single bisulfite accessible base pairs could arise for many different reasons during this analysis although, as can be seen in Fig 2, some recur and could be biologically interesting. Rather, consistent with previous studies, our algorithm predominantly identifies as important those bisulfite accessible sites containing two or more consecutive Cs that were converted to T by bisulfite as bisulfite accessible regions, or BARs. Under the assumption that each BAR is associated with a single polymerase complex, this result suggests, at least in Ramos V regions, that there are unlikely to be multiple polymerase complexes present on an individual V gene at any given time. Furthermore, the existence of a single BAR at recurring positions within the V region suggests that polymerase pausing is occurring in a significant subset of cells and that it may occur at specific positions along the V gene. Lower frequency BARs were observed throughout the V gene (Fig 2) which are presumably associated either with more transient or less recurrent ssDNA exposure.

While the size range of BARs is similar to that reported for transcription bubbles [33–35] (Fig 3C), we do not know if there is any relationship between the BARs and transcription bubbles. In fact, the BARs could also represent non-B DNA structures such as stem loops and I-motifs [57–60] that could also have an association between ssDNA secondary structure and accessibility within the chromatin to AID or other factors. In addition, highly stable DNA structures have been identified within 15 bp of transcription pausing sites genome wide [37]. We did search for potential stem loop structures which had been suggested to play a role in mutation [61] but found no association. Since it has recently been shown that G-quadruplexes (G4s) bind AID and play a role in recruiting it to switch regions to carry out class switch recombination [12], we used a pre-trained deep-learning based prediction method (G4detector) to predict the overall probability of G4 formation, followed by Integrated Gradients, a technique that enables mapping of the predicted overall G4 probability to specific input sites. In practice these input sites were almost always Gs, as one would expect. Surprisingly, there was a strong overlap between the positions of sites predicted to contribute to the G4 structure and BARs on the opposite strand, but not on the same strand (Fig 4). One interpretation of this result is that G4s may drive stable exposure of the complementary C sites (both within and around the G tract) on the opposite strand, thus making the ssDNA accessible (Fig 5D) [38, 62, 63]. An alternative potential explanation is that the G4 structure on the transcribed strand may cause transcription pausing or backtracking that produce recurring BARs in the corresponding subregion on the opposite strand of V gene.

Since previous papers had shown a rough positive correlation between BARs and SHM in whole exons but not at a base pair resolution, we further investigated the possible spatial association between AID mutations and the positioning of BARs. The nature of the assay makes it impossible to connect mutational events to accessibility directly (on the same DNA molecule), or indeed accessibility on both strands of the same dsDNA patch. Although AID induced V region mutation occurs at a relatively high frequency, the absolute numbers of mutations are still very low even in vivo and even lower in cell lines. The IGHV4–34*01 gene expressed in Ramos cell line mutates at a rate of 10^−5^/bp/generation [64] and there are very few sites in the IGHV gene in Ramos that are undergoing SHM at any moment, which further complicates the study of an association between BARs and SHM. However, it is almost impossible to collect enough primary germinal center B cells, therefore the Ramos cell line is still a suitable system to pursue this. Moreover, the IGHV4–34 gene is widely used in B cells in humans [65] which makes the Ramos cell line a good system to study the mechanistic regulation of SHM. Although we find no association between the SHM of all the sites and their bisulfite frequency (S2 Fig), we do observe a modest but significant correlation between the SHM of the most highly mutated sites and their bisulfite frequency site-to-site (Fig 5A). While a majority of the recurrently AID mutated sites do occur within 15bp of a BARs on either strand and in certain cases they are very close (≤5 bp) or overlap exactly (S3 Fig), we cannot exclude the possibility that this result is somewhat expected given that both BARs and highly mutated sites are widely distributed throughout the V gene. However, we do notice that the frequency of SHM for these highly mutated sites positively correlates with their distance to those BARs that are within 15 bp (Fig 5B and 5D). In order to mutate specific sites within IGHVs to increase antibody affinity, AID is not only recruited and stabilized at the IGHV region [66] but also tightly regulated by multiple factors including transcription complexes and factors that are involved in the processing of nascent RNA [2, 8] creating a crowded local environment rather than just naked ssDNA (Fig 5D). A biochemical study using human RNA polymerase suggests that although documented pause sites and AID mutations do not overlap, the sites frequently mutated by AID are around 15 bp from the core of transcription bubble [43]. Moreover, one computational study had shown that DNA G4 structure formation positively correlates with transcription pausing within 10–40 nt [37]. Based on these observations, SHM might be expected to be decreased close to the center of the BARs due to the decreased abundance of factors that can facilitate AID. In fact, previous papers had shown that AID targeting and mutation does not occur on the core of G4 structure in the switching region of Ig locus that is responsible to the change of antibody isotype, but rather on the adjacent ssDNA overhangs [67]. Similarly, here, we found the predicted G4 structure highly colocalized with BARs on the opposite strand of a human IGHV gene, and the sites highly mutated by AID mainly locate to the adjacent region which is around 15 bp from the core of the BARs on both strands (Fig 5D).

While we speculate that mutations at certain sites may depend on BAR formation, proving this will require extensive studies in which the sequences of individual bisulfite accessible regions are systematically mutated, and the frequency of AID and bisulfite accessible sites are analyzed in detail. At the very least, it is clear that greater temporal accessibility of ssDNA alone is probably not the major determinant of mutation [43], which in turn suggests that other regulatory mechanisms of AID must play important roles in determining whether mutations occur. For example, it has been suggested that AID interacts with Pol II and there is a “licensing” step for mutation associated with elongation after AID has be recruited to the chromatin [68].

## Materials and methods

### Cell culture and treatment

Rep161 cell line was generated using the human Burkitt’s lymphoma Ramos cell line [64]. Rep161 was maintained in Iscove’s modified Dulbecco’s medium supplemented with 10% FBS and 100 U/mL penicillin-streptomycin as described previously [7]. For somatic hypermutation induction, Rep161 was treated with 4-OHT (0.25 *μ*M) for 7 days [7].

### Bisulfite treatment and the IGHV4–34 region library preparation

Cells that were not treated with 4-OHT were collected after one week of culture and Bisulfite treatment was performed as previously [13]. Briefly, 10 million cells were fixed with 1% formaldehyde for 5 min at room temperature and the reaction was stopped with glycine to a final concentration of 125 mM. Then the nuclei were purified and permeabilized, followed by incubation in a fresh prepared solution containing 5 M sodium bisulfite and 20 mM hydroquinone for 18 h at 37°C. Finally, the nuclei were decross-linked and DNA was purified. For IGHV4–34 region, the primers (Fw: GTTGAAGCCTTCGGAGACCC, Rev: GGCAGTAGCAGAGAACAGAG) with UMI were used to amplify the V region from the genomic DNA. Then the final library was purified using QIAGEN GeneRead Size Selection Kit. The library was sequenced (2 × 300 bp) in MiSeq machine with 30% PhiX spike in using the v3 chemistry (300 cycles per end, 600 cycles total) by GENEWIZ. The IGHV4–34 region in Ramos cells has accumulate some mutations over the years and its sequence is not identical to the sequence in IMGT. Here we compare the sequence to that which is present in cells before and after treatment.

### Preparation of library for deep sequencing to identify AID induce somatic mutations

Cells were treated with 4-OHT for 7 days and genomic DNA was extracted using DNeasy Blood & Tissue Kits (QIAGEN). Then the IGHV4–34 region was amplified using primers (Fw: GTTGAAGCCTTCGGAGACCC, Rev: GGCAGTAGCAGAGAACAGAG) with UMI. The library was purified using GeneRead Size Selection Kit and then was sequenced at GENEWIZ using the MiSeq machine with the same parameters to the sequencing described above.

### Processing of deep sequencing dataset

For both Bisulfite and SHM deep sequencing data, the raw fastq data were processed using SHMprep (http://www.ams.sunysb.edu/∼maccarth/software.html) with default parameters and CONSCOUNT being set to 3. The output FASTA files were then processed to generate the mutation matrices for further analysis. Both the output FASTA files and the mutation matrices are included as S1 to S10 Datasets. For SHM, mutation rate calculations and SHM plots were done using R scripts (https://github.com/Jun2BCR/BCR_analysis).

### Clustering

Both top and bottom datasets consist of sequences with different bisulfite accessible regions. So we first divided them into groups so that sequences in each group all have the same bisulfite accessible structure. Clustering algorithms naturally serve this purpose. As the bisulfite accessible sites are binary (0 or 1), we used Hamming distance *d*(*x*, *y*) = *x* ⊕ *y* as the distance metric between sequences. As Hamming distance is not Euclidean, we chose complete-linkage clustering.

$$\begin{array}{c} D\left(X,Y\right)=max_{x\in X,y\in Y}d\left(x,y\right)\;\text{for}\;\text{two}\;\text{clusters}\;X,Y \end{array}$$

Picking the optimal number of clusters needs evidence from data and background information. Application of the gap statistic [69] suggested both the top strand and bottom strand datasets should be divided into 6 groups.

### Bisulfite data analysis using Bayesian model

Dividing the whole dataset into different clusters, we assume sequences in a cluster all have the same bisulfite accessible regions, and when they are added up, the sites are binomial distributed. Using the multiple change-point method [70, 71], we develop a Bayesian segmentation model to detect bisulfite accessible regions.

Consider a sequence of random variables $X_{\tau_{1}},X_{\tau_{2}},\ldots ,X_{\tau_{N}}$ indexed by locations {*τ*_*t*_}, and $X_{\tau_{t}}∼Binomial\left(\theta_{t},n\right)$. $\left\{X_{\tau_{t}}\right\}$ are independent given {*θ*_*t*_}. *θ*_*t*_ are not all the same, instead they are piecewise constant. For example, bisulfite accessible regions have a much higher *θ* than bisulfite inaccessible regions. So we have another independent sequence $I_{\tau_{1}},I_{\tau_{2}},\ldots ,I_{\tau_{N}}$, in which $I_{\tau_{t}}∼Bernoulli\left(p_{t}\right)$, indicating whether the parameter *θ* changed at *τ*_*t*_. Note that if $I_{\tau_{t}}=1$ then *τ*_*t*_ could be a boundary between bisulfite accessible and inaccessible regions, by convention $I_{\tau_{1}}=1$. Then *θ* holds constant until next change, that is *θ*_*t*_ = *θ*_*t*−1_ if $I_{\tau_{t}}=0$. Our Bayesian model has two sets of priors. First, there is a prior probability $p_{t}=Pr\left(I_{\tau_{t}}=1\right)$ for each *τ*_*t*_. Second, when $I_{\tau_{t}}=1$ the new *θ*_*t*_ is generated from the prior *Beta*(*μ*_0_, *ν*_0_) in mean and sample size form. We aim at deriving the posterior distributions $f(\theta_{t}|x_{\tau_{1}},x_{\tau_{2}},\ldots ,x_{\tau_{N}})$ and posterior probability of change $Pr(I_{\tau_{t}}=1|x_{\tau_{1}},x_{\tau_{2}},\ldots ,x_{\tau_{N}})$ for every *τ*_*t*_.

Given observed data $x_{\tau_{i}},\ldots ,x_{\tau_{j}}$ with length *m* and the sufficient statistic $X_{i:j}=x_{\tau_{i}}+\ldots +x_{\tau_{j}}$. If they share the same *θ* then in posterior the parameters are updated as *ν*_0_ → *ν*_*i*:*j*_, *μ*_0_ → *μ*_*i*:*j*_ and the normalizing constant updated as *c*_0_ → *c*_*i*,*j*_. So for any position *τ*_*t*_, suppose the segment containing *τ*_*t*_ starts from *τ*_*i*_ and ends at *τ*_*j*_, that is $I_{\tau_{i}}=1,I_{\tau_{j+1}}=1$ and every indicator between them are all 0, then we already have everything for the posterior $f(\theta_{t}|X_{i:j})=Beta(\theta_{t}|\mu_{i:j},\nu_{i:j})$ are unknown. If we know, conditional on the data, the probabilities $w_{i,j,t}=Pr\left(I_{\tau_{i}}=1\cap I_{\tau_{j+1}}=1\cap I_{\tau_{k}}=0,i<k\leq j|x_{\tau_{1}},x_{\tau_{2}},\ldots ,x_{\tau_{N}}\right)$ for any 1 ≤ *i* ≤ *t* ≤ *j* ≤ *N*, then we can write the posterior as:

$$\begin{array}{c} f\left(\theta_{t}|X_{1:N}\right)=\sum_{1\leq i\leq t\leq j\leq N}w_{i,j,t}Beta\left(\theta_{t}|\mu_{i:j},\nu_{i:j}\right) \end{array}$$

Now the problem is how to calculate $\left\{w_{i,j,t}\right\}$. Notice that, we can also decompose $f(\theta_{t}|X_{1:N})$ as (see S1 Method for the details):

$$\begin{array}{c} f(\theta_{t}|X_{1:N})\propto \frac{f\left(\theta_{t}|X_{1:t}\right)f\left(\theta_{t}|X_{t+1:N}\right)}{Beta(\theta_{t}|\nu_{0},\mu_{0})} \end{array}$$ (0.1)

So we shall start with $f(\theta_{t}|X_{1:t})$ and $f(\theta_{t}|X_{t+1:N})$.

#### Calculating posteriors

Let $p_{i,t}=Pr\left(k_{t}=i|X_{1:t}\right)$ denote, conditional on $X_{1:t}$, the probability that *τ*_*i*_ ≤ *τ*_*t*_ is the most recent change up to *τ*_*t*_. Then we can decompose $f(\theta_{t}|X_{1:t})$ as

$$\begin{array}{cc} f(\theta_{t}|X_{1:t}) & =\sum_{i=1}^{t}p_{i,t}f\left(\theta_{t}|k_{t}=i,X_{1:t}\right)\\ & =\sum_{i=1}^{t}p_{i,t}Beta\left(\theta_{t}|\mu_{i,t},\nu_{i,t}\right) \end{array}$$

We have the recursive equation (see S1 Method for the details) and $\sum_{i=1}^{t}p_{i,t}=1$ to calculate $\left\{p_{i,t}\right\}$.

$$\begin{array}{c} p_{i,t}\propto p_{i,t}^{⋆}=\{\begin{array}{cc} p_{t}\frac{c_{0}}{c_{t,t}}, & i=t\\ \left(1-p_{t}\right)p_{i,t-1}\frac{c_{i,t-1}}{c_{i,t}}, & i<t \end{array} \end{array}$$ (0.2)

Let $q_{j,t+1}=Pr\left({\tilde{k}}_{t+1}=j|X_{t+1:N}\right)$ denote, conditional on $X_{t+1:N}$, the probability that *τ*_*j*_ ≥ *τ*_*t*_ is the last data point before a new change at *τ*_*j*+1_. Then we can decompose $f(\theta_{t}|X_{t+1:N})$ in a similar way.

$$\begin{array}{cc} f(\theta_{t}|X_{t+1:N}) & =\sum_{j=t}^{N}q_{j,t+1}f\left(\theta_{t}|{\tilde{k}}_{t+1}=j,X_{t+1:N}\right)\\ & =q_{t,t+1}Beta\left(\theta_{t}|\mu_{0},\nu_{0}\right)+\sum_{j=t+1}^{N}q_{j,t+1}Beta\left(\theta_{t}|\mu_{t+1:j},\nu_{t+1:j}\right) \end{array}$$

Where $q_{t,t+1}=p_{t+1}$. We have the recursive equation (see S1 Method for the details) and $\sum_{j=t}^{n}q_{j,t+1}=1$ to calculate $\left\{q_{j,t+1}\right\}$.

$$\begin{array}{c} q_{j,t+1}\propto q_{j,t+1}^{⋆}=\{\begin{array}{cc} \left(1-p_{t+1}\right)p_{t+2}\frac{c_{0}}{c_{t+1,t+1}}, & j=t+1\\ \left(1-p_{t+1}\right)q_{j,t+2}\frac{c_{t+2,j}}{c_{t+1,j}}, & j>t+1 \end{array} \end{array}$$ (0.3)

Now we have all the components for Eq 0.1.

$$\begin{array}{c} f\left(\theta_{t}|X_{1:N}\right)\propto \frac{f\left(\theta_{t}|X_{1:t}\right)f\left(\theta_{t}|X_{t+1:N}\right)}{Beta(\theta |\mu_{0},\nu_{0})}\\ =\sum_{i=1}^{t}p_{i,t}q_{t,t+1}Beta\left(\theta_{t}|\mu_{i:t},\nu_{i:t}\right)+\sum_{i=1,j=t+1}^{i=t,j=N}p_{i,t}q_{j,t+1}\frac{Beta\left(\theta_{t}|\mu_{i:t},\nu_{i:t}\right)Beta\left(\theta_{t}|\mu_{t+1:j},\nu_{t+1:j}\right)}{Beta(\theta_{t}|\mu_{0},\nu_{0})} \end{array}$$

So we have the recursive equation (see S1 Method for the details) and $\sum_{1\leq i\leq t\leq j\leq N}\;w_{i,j,t}=1$ to calculate $\left\{w_{i,j,t}\right\}$.

$$\begin{array}{c} w_{i,j,t}\propto w_{i,j,t}^{⋆}=\{\begin{array}{cc} p_{i,t}q_{t,t+1}, & i\leq t=j\\ p_{i,t}q_{j,t+1}\frac{c_{i,t}c_{t+1,j}}{c_{i,j}c_{0}}, & i\leq t<j \end{array} \end{array}$$

$$\begin{array}{c} w_{i,j,t}=\frac{w_{i,j,t}^{⋆}}{\sum_{1\leq i\leq t\leq j\leq N}w_{i,j,t}^{⋆}} \end{array}$$ (0.4)

Most importantly, the posterior mean of {*θ*_*t*_} is

$$\begin{array}{c} E\left(\theta_{t}|X_{1:N}\right)=\sum_{1\leq i\leq t\leq j\leq N}w_{i,j,t}\mu_{i:j} \end{array}$$ (0.5)

Based on everything we have, the posterior probability of change $Pr(I_{\tau_{t}}=1|X_{\tau_{1}},X_{\tau_{2}},\ldots ,X_{\tau_{N}})$ is

$$\begin{array}{cc} Pr(I_{\tau_{t+1}}=1|X_{1:N}) & =\frac{p_{t+1}}{\sum_{1\leq i\leq t\leq j\leq N}\;w_{i,j,t}^{⋆}} \end{array}$$ (0.6)

#### Hyperparameters

As a Bayesian method, we need to specify the hyperparameters. We take an empirical Bayesian approach so the hyperparameters are estimated from data. For prior sample size *ν*_0_, our suggestion is to set *ν*_0_ = *n*. As $X_{\tau_{t}}∼Binomial\left(\theta_{t},n\right)$, it can be interpreted as the prior stands for one data point. For *μ*_0_, we take it as $\mu_{0}=\sum_{1}^{N}x_{\tau_{t}}/\left(Nn\right)$. So the prior mean is just the sample mean.

The prior probability of change at each *τ*_*t*_ is a little more complicated. In bisulfite data the spaces between adjacent *τ*_*t*_’s are not constant, that is *τ*_*t*+1_ − *τ*_*t*_ is not always the same. This is because the nucleobases C or G are not evenly distributed along DNA. If *τ*_*t*_ − *τ*_*t*−1_ is large, then there is more space for changes to occur in between *τ*_*t*−1_ and *τ*_*t*_. The occurance of changes is a Poisson process with rate λ. So we have:

$$\begin{array}{c} p_{t}=\{\begin{array}{cc} 1, & t=1\\ 1-e^{-\lambda (\tau_{t}-\tau_{t-1})}, & t>1 \end{array} \end{array}$$

The value of λ is taken to maximize the marginal density of the whole sequence (see S1 Method for the details)

$$\begin{array}{c} f\left(x_{\tau_{1}},x_{\tau_{2}},\ldots ,x_{\tau_{N}}\right)=\prod_{t=1}^{N}(\sum_{i=1}^{t}p_{i,t}^{⋆}) \end{array}$$ (0.7)

This optimization can be solved by grid search.

Another point is that different groups have different values of *n* in $X_{\tau_{t}}∼Binomial\left(\theta_{t},n\right)$, so we need to scale them to be equal. For the reason, consider the posterior mean and variance of $f(\theta_{t}|X_{\tau_{1}},X_{\tau_{2}},\ldots ,X_{\tau_{N}})$.

Setting *ν*_0_ = *n*, the posterior updates are

$$\begin{array}{c} \nu_{0}\to \nu_{i:j}=n+mn\\ \mu_{0}\to \mu_{i:j}=\frac{n\mu_{0}+X_{i:j}}{n+mn} \end{array}$$

The posterior mean *μ*_*i*:*j*_ remains the same if we divide $\left\{X_{\tau_{t}}\right\}$ and *n* by any constant. But posterior variance would change.

$$\begin{array}{cc} Var_{i:j} & =\frac{\left(n\mu_{0}+X_{i:j}\right)\left(n-n\mu_{0}-X_{i:j}\right)}{n^{2}\left(1+n\right)}\\ & =\frac{\left(\mu_{0}+X_{i:j}/n\right)\left(1-\mu_{0}-X_{i:j}/n\right)}{1+n} \end{array}$$

The larger the *n*, the smaller the posterior variance of *θ*_*t*_.

#### Patch size and distance calculation

Our method can capture continuous changes at the boundaries of a BAR, so we output a distribution of the BAR size and take its expectation, as shown in Fig 3. The set of $\left\{w_{i,j,t}\right\}$ is the distribution of patch containing position *τ*_*t*_. So the average bisulfite accessible patch size for a BAR can be calculated by $\left\{w_{i,j,t}\right\}$ for the peak mutation site *τ*_*t*_ of that cluster.

### DNA G-quadruplex structure prediction

We used G4detector to estimate the probability of a sequence to form a G-quadruplex. G4detector is a deep learning model that accepts DNA sequences of 297 nts in its one-hot encoding format (i.e. a matrix of 0’s and 1’s) as input, and outputs a single number representing the G4 potential of the sequence. In order to approximate the G4 potential of the longer Ramos IGHV4–34 V region, which contains 346 nts, we calculated the G4 potential of the sequence at various windows of size 297 nts, starting at the beginning of the sequence, and then moving over 1 bp until the end of the sequence was reached. To assess the bottom strand sequence predictions, we took the reverse complement of the top strand, and repeated the same procedure as we did for the top strand.

### Finding G-repeats

We utilized Integrated Gradients to identify G-repeats within a sequence that were suspected to be involved in forming the predicted G-quadruplex since G4detector does not reveal that information directly. Integrated Gradients is an attribution method that works by taking the straight-line path integral from some baseline reference (e.g. 0-matrix), to the input. In other words, we use Integrated Gradients in order to map the prediction of G4detector to the relevant input features. Since we utilize G4detector to estimate the G4 potential of the IGHV4–34 gene at multiple frames of the sequences, we averaged the contribution scores outputted by Integrated Gradients. To calculate the contribution score at a site, we summed over the scores at a site and then divided by the number of times we observed an overlap at that site.

### Calculation of distance between the highly mutated sites and the BARs from both strands

For each of the top 25 highly mutated sites from both strands, we first calculated the signed distance between this site to all the sites with each BAR that are determined by our Bayesian Segmentation model. For each site in the BAR, there is a “posterior response probability to bisulfite” estimated by our model shown by red dots in Fig 2A and 2C. Second, we multiplied the distance between the mutation site and each site in the BAR with their respective “posterior response probability to bisulfite” to calculate the average value. The final weighted averages are the distance numbers shown in S3 Fig.

## Supporting information

S1 Fig Comparison of SHM for IGHV4–34 gene from both Rep161 cell line and the primary human B cells. (A) Comparison of distribution pattern of SHM for IGHV4–34 gene from both Rep161 and primary human B cells. x-axis shows the nucleotide position of IGHV4–34 gene and the y-axis shows the mutation frequency for each site in the form of 10^−3^ for Rep161 (due to low mutation frequency in cell line) and 10^−1^ for Human primary B cells. CDR1 and CDR2 regions in IGHV4–34 gene are labeled with gray background. (B) the site-to-site correlation of SHM for IGHV4–34 between Rep161 in the upper panel and primary human B cells in the lower panel. The values for each site from both Rep161 and primary human B cells (as shown in panel A) are transformed to Log2 form for better visualization. Red dots represent each nucleotide position. Blue line shows the regression line and the gray shadow represents the corresponding 95% confidence area. Human_B_SHM means the frequency of SHM in human primary B cells.
(TIF)

S2 Fig Determination of direct correlation between SHM and the BARs. (A) The mutation rate for bisulfite conversion and SHM. The top panel shows the frequency of mutation in each C (top strand shown in red vertical line) or G (bottom strand shown in black vertical line) site by bisulfite conversion. The bottom panel shows the SHM in each C or G site by AID (activation-induced deaminase). X-axis is the nucleotide position of IGHV4–34 gene and Y-axis represents the mutation rate of each nucleotide position. The black arrows indicate the nucleotide positions that are mentioned in the corresponding part of “Results” section. (B) the correlation between SHM in the top strand and the BARs in the top strand. (C) the correlation between SHM in the bottom strand and the BARs in the bottom strand. (D) the correlation between SHM in the top strand and the BARs in the bottom strand. (E) the correlation between SHM in the bottom strand and the BARs in the top strand. For each correlation analysis, the R and the p value is shown in the plot. Red dots represent each nucleotide position. Blue line represents the regression line and the gray shadow represents the corresponding 95% confidence area.
(TIF)

S3 Fig Determination of direct correlation between SHM and the BARs. “Top 25 sites of C-SHM” indicates the top 25 highly mutated C sites of SHM in top strand and the “Top 25 sites of G-SHM” indicates bottom strand. For each table, the first row shows the BARs clusters in each strand, the first column shows the motifs where the C (red bold nucleotide in motifs) is mutated and the second column displays the position of the mutated C and the position of the Cs is ordered by the frequency of SHM in each site in descending order. The number in each table is the pairwise base-pair distance between the mutation site and the BAR. Setting 15 bp as a threshold based on the size of transcription and the average size of the patch in BARs, the numbers with a gray color background are within the threshold.
(TIF)

S1 Method Detailed information on the derivation of equations. (PDF)

S1 Dataset DNA sequences (FASTA format) from bisulfite assay from Rep161 cells without 4-OHT treatment. (FASTA)

S2 Dataset Matrices of bisulfite accessible sites in top strand from S1 Dataset. (TXT)

S3 Dataset Matrices of bisulfite accessible sites in bottom strand from S1 Dataset. (TXT)

S4 Dataset DNA sequences (FASTA format) of background SHM from Rep161 cells without 4-OHT treatment. (FASTA)

S5 Dataset Matrices of background SHM in top strand from S4 Dataset. (TXT)

S6 Dataset Matrices of background SHM in bottom strand from S4 Dataset. (TXT)

S7 Dataset DNA sequences (FASTA format) of SHM from Rep161 cells with 4-OHT treatment. (FASTA)

S8 Dataset Matrices of SHM in top strand from S7 Dataset. (TXT)

S9 Dataset Matrices of SHM in bottom strand from S7 Dataset. (TXT)

S10 Dataset Frequency (DUPCOUNT) of each sequence in S2, S3, S5, S6, S8 and S9 Datasets. (TXT)
