# Supplementary figures and images for: A Bayesian model based computational analysis of the relationship between bisulfite accessible single-stranded DNA in chromatin and somatic hypermutation of immunoglobulin genes
Source: PLoS Comput Biol. 2021 Sep 7;17(9):e1009323. doi: 10.1371/journal.pcbi.1009323 (PMC8462741; doi:10.1371/journal.pcbi.1009323)

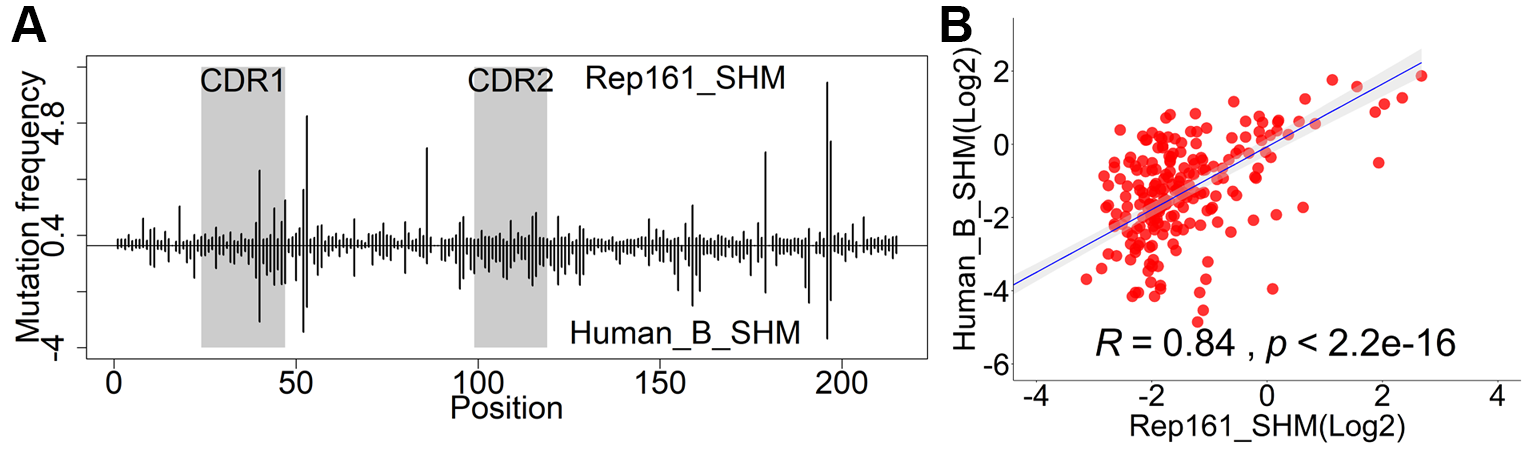

Supplement: S1 Fig — (A) Comparison of distribution pattern of SHM for IGHV4–34 gene from both Rep161 and primary human B cells. x-axis shows the nucleotide position of IGHV4–34 gene and the y-axis shows the mutation frequency for each site in the form of 10−3 for Rep161 (due to low mutation frequency in cell line) and 10−1 for Human primary B cells. CDR1 and CDR2 regions in IGHV4–34 gene are labeled with gray background. (B) the site-to-site correlation of SHM for IGHV4–34 between Rep161 in the upper panel and primary human B cells in the lower panel. The values for each site from both Rep161 and primary human B cells (as shown in panel A) are transformed to Log2 form for better visualization. Red dots represent each nucleotide position. Blue line shows the regression line and the gray shadow represents the corresponding 95% confidence area. Human_B_SHM means the frequency of SHM in human primary B cells. (TIF) [file pcbi.1009323.s001.tif]

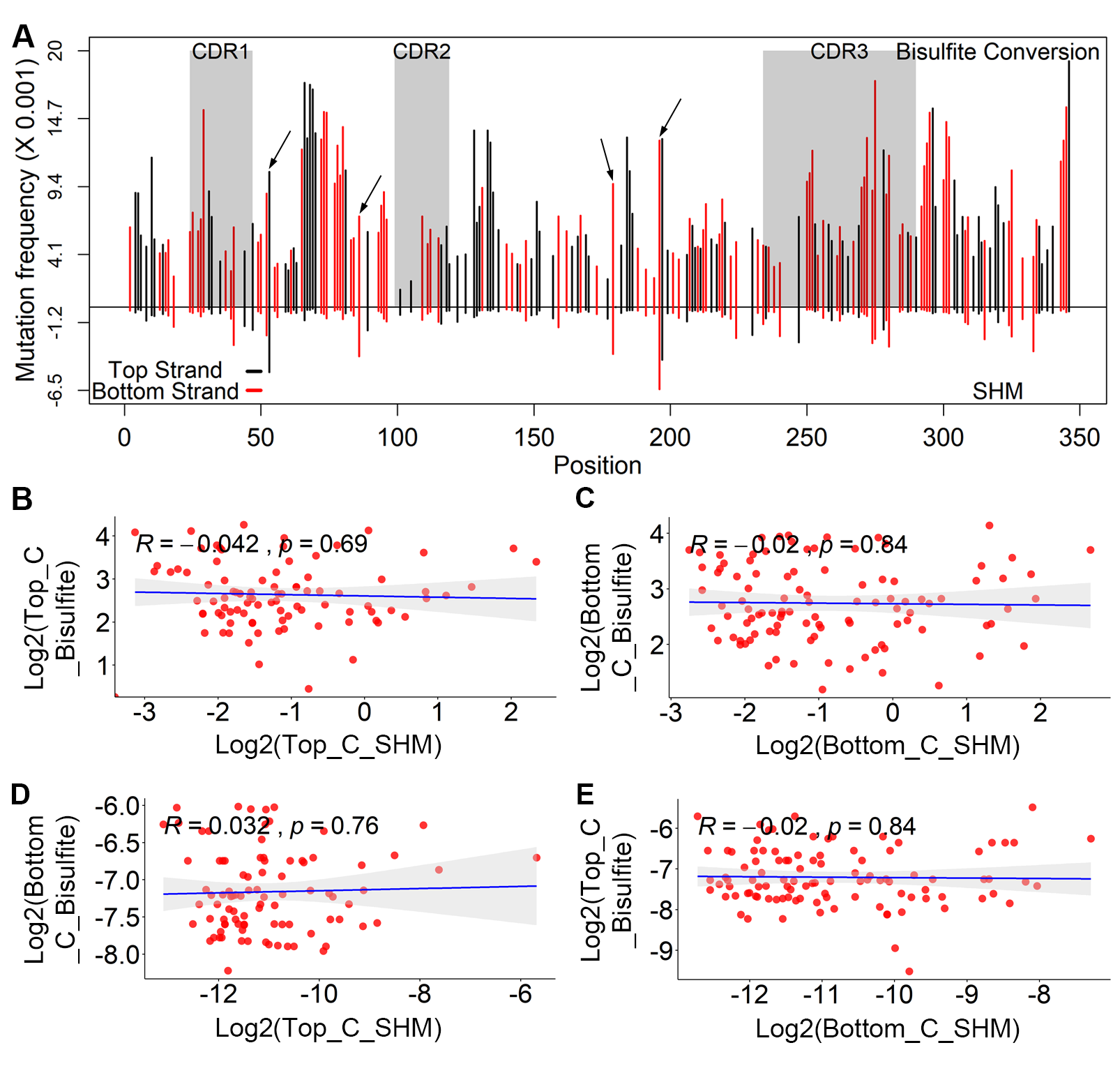

Supplement: S2 Fig — (A) The mutation rate for bisulfite conversion and SHM. The top panel shows the frequency of mutation in each C (top strand shown in red vertical line) or G (bottom strand shown in black vertical line) site by bisulfite conversion. The bottom panel shows the SHM in each C or G site by AID (activation-induced deaminase). X-axis is the nucleotide position of IGHV4–34 gene and Y-axis represents the mutation rate of each nucleotide position. The black arrows indicate the nucleotide positions that are mentioned in the corresponding part of “Results” section. (B) the correlation between SHM in the top strand and the BARs in the top strand. (C) the correlation between SHM in the bottom strand and the BARs in the bottom strand. (D) the correlation between SHM in the top strand and the BARs in the bottom strand. (E) the correlation between SHM in the bottom strand and the BARs in the top strand. For each correlation analysis, the R and the p value is shown in the plot. Red dots represent each nucleotide position. Blue line represents the regression line and the gray shadow represents the corresponding 95% confidence area. (TIF) [file pcbi.1009323.s002.tif]

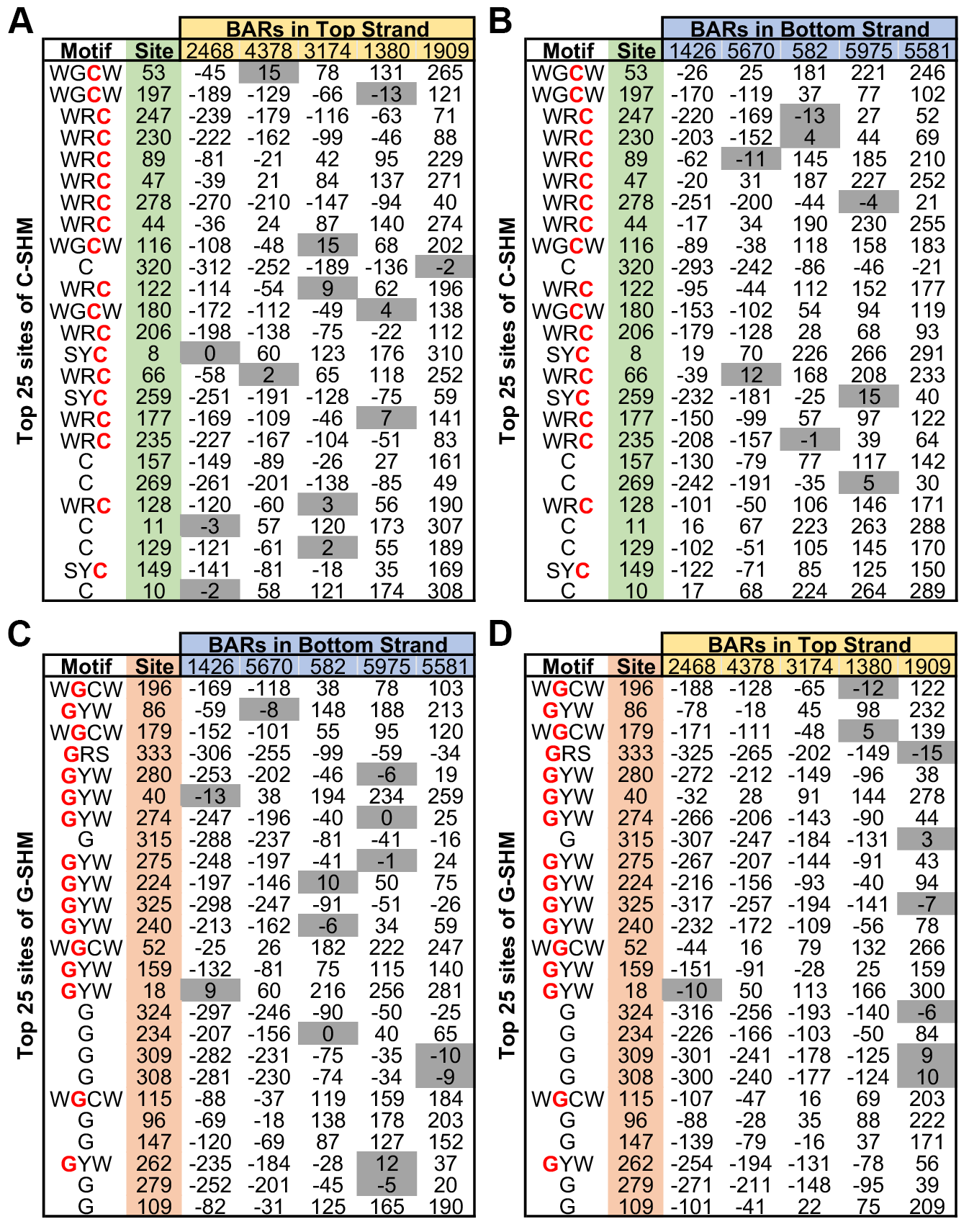

Supplement: S3 Fig — “Top 25 sites of C-SHM” indicates the top 25 highly mutated C sites of SHM in top strand and the “Top 25 sites of G-SHM” indicates bottom strand. For each table, the first row shows the BARs clusters in each strand, the first column shows the motifs where the C (red bold nucleotide in motifs) is mutated and the second column displays the position of the mutated C and the position of the Cs is ordered by the frequency of SHM in each site in descending order. The number in each table is the pairwise base-pair distance between the mutation site and the BAR. Setting 15 bp as a threshold based on the size of transcription and the average size of the patch in BARs, the numbers with a gray color background are within the threshold. (TIF) [file pcbi.1009323.s003.tif]
