# Supplementary material for: A Bayesian model based computational analysis of the relationship between bisulfite accessible single-stranded DNA in chromatin and somatic hypermutation of immunoglobulin genes
Source: PLoS Comput Biol. 2021 Sep 7;17(9):e1009323. doi: 10.1371/journal.pcbi.1009323 (PMC8462741; doi:10.1371/journal.pcbi.1009323)
Supplement: S1 Method — (PDF) [file pcbi.1009323.s004.pdf]

Supporting Information File1: A Bayesian model based computational analysis of the relationship between bisulfite accessible single-stranded DNA in chromatin and somatic hypermutation of Immunoglobulin genes

February 20, 2021

1 Derivation of Equations

The prior distribution $Beta(\mu_0, \nu_0)$ in mean and sample size form with ν_0 stands for prior sample size and μ_0 stands for prior mean.

$$
\begin{aligned}
Beta(\theta|\mu_0, \nu_0) &= \frac{\exp\left\{(\nu_0\mu_0 - 1)\log\theta + (\nu_0 - \nu_0\mu_0 - 1)\log(1-\theta)\right\}}{B(\nu_0\mu_0, \nu_0 - \nu_0\mu_0)} \\
c_0 &= \frac{1}{B(\nu_0\mu_0, \nu_0 - \nu_0\mu_0)}
\end{aligned}
$$

Where c_0 is the normalizing constant. Posterior updates:

$$
\begin{aligned}
\nu_0 &\to \nu_{i:j} = \nu_0 + mn \\
\mu_0 &\to \mu_{i:j} = \frac{\nu_0\mu_0 + \mathscr{X}_{i:j}}{\nu_0 + mn} \\
c_0 &\to c_{i,j} = \frac{1}{B(\nu_0\mu_0 + \mathscr{X}_{i:j}, \nu_0 + mn - \nu_0\mu_0 - \mathscr{X}_{i:j})}
\end{aligned}
$$

According to our model we have the marginal distribution of X_{τ_t} given any μ and ν

$$\int f(x|\theta)Beta(\theta|\mu,\nu)d\theta = \frac{B(\nu\mu + x, \nu + n - \nu\mu - x)}{B(\nu\mu, \nu - \nu\mu)} \tag{1.1}$$

Equation (0.1) Decomposition of $f(\theta_t|\mathscr{X}_{1:N})$

$$\begin{aligned}
f(\theta_t|\mathscr{X}_{1:N}) &\propto f(\theta_t, \mathscr{X}_{1:N}) \\
&= f(\theta_t, \mathscr{X}_{1:t}, \mathscr{X}_{t+1:N}) \\
&= f(\mathscr{X}_{t+1:N}|\theta_t, \mathscr{X}_{1:t})f(\theta_t, \mathscr{X}_{1:t}) \\
&= f(\mathscr{X}_{t+1:N}|\theta_t)f(\mathscr{X}_{1:t}, \theta_t) \\
&= \frac{f(\theta_t|\mathscr{X}_{1:t})f(\theta_t|\mathscr{X}_{t+1:N})}{f(\theta_t)} \\
&\propto \frac{f(\theta_t|\mathscr{X}_{1:t})f(\theta_t|\mathscr{X}_{t+1:N})}{f(\theta_t)} \\
&= \frac{f(\theta_t|\mathscr{X}_{1:t})f(\theta_t|\mathscr{X}_{t+1:N})}{Beta(\theta_t|\nu_0, \mu_0)}
\end{aligned}$$

Equation (0.2) Calculation of $\{\mathfrak{p}_{i,t}\}$

$$\begin{aligned}
\mathfrak{p}_{t,t} \propto Pr(k_t = t, \mathscr{X}_{1:t}) &= Pr(k_t = t, x_{\tau_t}, \mathscr{X}_{1:t-1}) \\
&= f(x_{\tau_t}|k_t = t, \mathscr{X}_{1:t-1})Pr(k_t = t|\mathscr{X}_{1:t-1})f(\mathscr{X}_{1:t-1}) \\
&= f(x_{\tau_t}|I_{\tau_t} = 1)p_t f(\mathscr{X}_{1:t-1})
\end{aligned}$$

And for $i < t$

$$
\begin{aligned}
\mathfrak{p}_{i,t} \propto Pr(k_t = i, \mathscr{X}_{1:t}) &= Pr(k_t = i, x_{\tau_t}, \mathscr{X}_{1:t-1}) \\
&= f(x_{\tau_t}|k_t = i, \mathscr{X}_{1:t-1})Pr(k_t = i|\mathscr{X}_{1:t-1})f(\mathscr{X}_{1:t-1}) \\
&= f(x_{\tau_t}|k_t = i, \mathscr{X}_{1:t-1})Pr(k_{t-1} = i, I_{\tau_t} = 0|\mathscr{X}_{1:t-1})f(\mathscr{X}_{1:t-1}) \\
&= f(x_{\tau_t}|k_t = i, \mathscr{X}_{1:t-1})Pr(I_{\tau_t} = 0|\mathscr{X}_{1:t-1})Pr(k_{t-1} = i|\mathscr{X}_{1:t-1})f(\mathscr{X}_{1:t-1}) \\
&= f(x_{\tau_t}|k_t = i, \mathscr{X}_{1:t-1})(1 - p_t)\mathfrak{p}_{i,t-1}f(\mathscr{X}_{1:t-1})
\end{aligned}
$$

In summary

$$
\mathfrak{p}_{i,t} \propto \mathfrak{p}^{\star}_{i,t} = \begin{cases} p_t f(x_{\tau_t}|I_{\tau_t} = 1), & i = t \\ (1 - p_t)\mathfrak{p}_{i,t-1} f(x_{\tau_t}|k_t = i, \mathscr{X}_{1:t-1}), & i < t \end{cases}
$$

Plug in the above $f(x_{\tau_t}|I_{\tau_t} = 1)$ and $f(x_{\tau_t}|k_t = i, \mathscr{X}_{1:t-1})$ with (1.1), in which μ and ν to be the corresponding posterior values. This leads to the recursive equation (0.2).

Equation (0.3) Calculation of $\{\mathfrak{q}_{j,t+1}\}$

$$
\mathfrak{q}_{t,t+1} = Pr(I_{\tau_{t+1}} = 1|\mathscr{X}_{t+1:N}) = p_{t+1} \tag{1.2}
$$

And for $j > t$

$$
\begin{aligned}
\mathfrak{q}_{j,t+1} \propto Pr(\widetilde{k}_{t+1} = j, \mathscr{X}_{t+1:N}) &= Pr(\widetilde{k}_{t+1} = j, x_{\tau_{t+1}}, \mathscr{X}_{t+2:N}) \\
&= f(x_{\tau_{t+1}}|\widetilde{k}_{t+1} = j, \mathscr{X}_{t+2:N})Pr(\widetilde{k}_{t+1} = j|\mathscr{X}_{t+2:N})f(\mathscr{X}_{t+2:N}) \\
&= f(x_{\tau_{t+1}}|\widetilde{k}_{t+1} = j, \mathscr{X}_{t+2:N})Pr(\widetilde{k}_{t+2} = j, I_{\tau_{t+1}} = 0|\mathscr{X}_{t+2:N})f(\mathscr{X}_{t+2:N}) \\
&= f(x_{\tau_{t+1}}|\widetilde{k}_{t+1} = j, \mathscr{X}_{t+2:N})Pr(I_{\tau_{t+1}} = 0|\mathscr{X}_{t+2:N})Pr(\widetilde{k}_{t+2} = j|\mathscr{X}_{t+2:N}). \\
&= f(x_{\tau_{t+1}}|\widetilde{k}_{t+1} = j, \mathscr{X}_{t+2:N})(1 - p_{t+1})\mathfrak{q}_{j,t+2}f(\mathscr{X}_{t+2:N})
\end{aligned}
$$

$$
\mathfrak{q}_{j,t+1} \propto \mathfrak{q}^{\star}_{j,t+1} = (1 - p_{t+1})\mathfrak{q}_{j,t+2} f(x_{\tau_{t+1}}|\widetilde{k}_{t+1} = j, \mathscr{X}_{t+2:N})
$$

That leads to the recursive Equation (0.3).

Equation (0.4) and (0.6) Posterior estimations

$$
\begin{aligned}
f(\theta_t|\mathscr{X}_{1:N}) &\propto \frac{f(\theta_t|\mathscr{X}_{1:t})f(\theta_t|\mathscr{X}_{t+1:N})}{Beta(\theta|\mu_0,\nu_0)} \\
&= \frac{\left\{\sum_{i=1}^{t}\mathfrak{p}_{i,t}Beta(\theta_t|\mu_{i:t},\nu_{i:t})\right\}\left\{\mathfrak{q}_{t,t+1}Beta(\theta_t|\mu_0,\nu_0)+\sum_{j=t+1}^{N}\mathfrak{q}_{j,t+1}Beta(\theta_t|\mu_{t+1:j},\nu_{t+1:j})\right\}}{Beta(\theta_t|\mu_0,\nu_0)} \\
&= \sum_{i=1}^{t}\mathfrak{p}_{i,t}\mathfrak{q}_{t,t+1}Beta(\theta_t|\mu_{i:t},\nu_{i:t}) + \sum_{i=1,j=t+1}^{i=t,j=N}\mathfrak{p}_{i,t}\mathfrak{q}_{j,t+1}\frac{Beta(\theta_t|\mu_{i:t},\nu_{i:t})Beta(\theta_t|\mu_{t+1:j},\nu_{t+1:j})}{Beta(\theta_t|\mu_0,\nu_0)}
\end{aligned}
$$

Where

$$
\begin{aligned}
&\frac{Beta(\theta_t|\mu_{i:t},\nu_{i:t})Beta(\theta_t|\mu_{t+1:j},\nu_{t+1:j})}{Beta(\theta_t|\mu_0,\nu_0)} \\
&= \frac{c_{i,t}c_{t+1,j}}{c_{i,j}c_0}Beta(\theta|\mu_{i:j},\nu_{i:j})
\end{aligned}
$$

Compare it with $f(\theta_t|\mathscr{X}_{1:N}) = \sum_{1\le i\le t\le j\le N}\mathfrak{w}_{i,j,t}Beta(\theta_t|\mu_{i:j},\nu_{i:j})$, we get Equation (0.4).

$$
\begin{aligned}
Pr(I_{\tau_{t+1}}=1|\mathscr{X}_{1:N}) &= \sum_{1\le i\le t=j\le N}\mathfrak{w}_{i,j,t} \\
&= \frac{\sum_{i\le t}\mathfrak{p}_{i,t}\mathfrak{q}_{t,t+1}}{\sum_{1\le i\le t\le j\le N}\mathfrak{w}^{\star}_{i,j,t}} \\
&= \frac{p_{t+1}}{\sum_{1\le i\le t\le j\le N}\mathfrak{w}^{\star}_{i,j,t}}
\end{aligned}
$$

Because $\mathfrak{q}_{t,t+1}=p_{t+1}$ (1.2) and $\sum_{i\le t}\mathfrak{p}_{i,t}=1$, we get Equation (0.6).

Equation (0.7) Marginal density of the whole sequence

First the one step ahead prediction density is

$$f(x_{\tau_t}|\mathscr{X}_{1:t-1}) = \sum_{i=1}^{t} Pr(k_t = i|\mathscr{X}_{1:t-1}) f(x_{\tau_t}|k_t = i, \mathscr{X}_{1:t-1}) = \sum_{i=1}^{t} \mathfrak{p}^{\star}_{i,t}$$

So the marginal density of the whole sequence is

$$f(x_{\tau_1}, x_{\tau_2}, ..., x_{\tau_N}) = \prod_{t=1}^{N} f(x_{\tau_t}|x_{\tau_1}, x_{\tau_2}, ..., x_{\tau_{t-1}}) = \prod_{t=1}^{N} (\sum_{i=1}^{t} \mathfrak{p}^{\star}_{i,t})$$
